# Berberine inhibits adipocyte differentiation, proliferation and adiposity through down-regulating galectin-3

**DOI:** 10.1038/s41598-019-50103-5

**Published:** 2019-09-16

**Authors:** Can Wang, Yan Wang, Shu-Rong Ma, Zeng-Yan Zuo, Yan-Bin Wu, Wei-Jia Kong, Ai-Ping Wang, Jian-Dong Jiang

**Affiliations:** 1 New Drug Safety Evaluation Center, Institute of Materia Medica, Chinese Academy of Medical Sciences and Peking Union Medical College, Beijing, China; 2 State Key Laboratory of Bioactive Natural Products and Function, Institute of Materia Medica, Chinese Academy of Medical Sciences and Peking Union Medical College, Beijing, China; 3 Department of Virology & NHC Key Laboratory of Biotechnology of Antibiotics, Institute of Medicinal Biotechnology, Chinese Academy of Medical Sciences and Peking Union Medical College, Beijing, China

**Keywords:** Molecular medicine, Target identification

## Abstract

This study is designed to investigate the effects of berberine (BBR) on galectin-3 (Gal-3) and the relationships to its suppressive activities on adipocyte differentiation, proliferation and adiposity. Our results showed that BBR greatly suppressed the differentiation and proliferation of mouse primary preadipocytes isolated from epididymal white adipose tissue (eWAT), during which the expression level of Gal-3 was down-regulated significantly. Overexpression of Gal-3 totally abolished the suppressive activities of BBR on Gal-3 expression, preadipocyte differentiation and proliferation. BBR reduced Gal-3 promoter activity, destabilized its mRNA and inhibited firefly luciferase activity of a recombinant plasmid containing the Gal-3 3′ untranslated region (UTR). Furthermore, BBR up-regulated microRNA (miRNA) let-7d expression and the suppressive activity on Gal-3 3′UTR was abolished by point mutation on the let-7d binding site. In mice fed a high-fat diet (HFD), BBR up-regulated let-7d and down-regulated Gal-3 expression in eWAT; it also suppressed adipocyte differentiation and proliferation and reduced adiposity greatly. In summary, our study proves that BBR inhibits the differentiation and proliferation of adipocytes through down-regulating Gal-3, which is closely associated with its anti-obesity effect. Our results may support the future clinical application of BBR for the treatment of obesity or related diseases.

## Introduction

The excessive differentiation or proliferation of adipocytes will cause an increase of their size or number, an expansion of adipose tissue, and obesity^1^. The prevalence of obesity has increased worldwide and obesity will increase the risks of metabolic diseases, cardiovascular diseases, and cancer^2–4^. Therefore, the research and development of novel anti-obesity drugs are of significant scientific as well as clinical importance.

The differentiation and proliferation of adipocytes are modulated by a series of factors or molecules, which include galectin-3 (Gal-3), a member of the family of Gals which bind carbohydrates or glycoproteins^5,6^. Gal-3 is expressed in various cell types and is secreted into extracellular fluid^5,6^. Gal-3 has a variety of biological activities which depend on different subcellular localization. For example, extracellular Gal-3 modulates interactions between cells; Gal-3 in cytoplasm is related to cell survival and death, while Gal-3 in nucleus may influence gene expression^5,6^.

Gal-3 is positively associated with obesity. In obese adults or children, the serum level of Gal-3 (which may reflect its secretion from cells) was found to be elevated, and this elevation was related to an increase of body fat^7,8^. In mice with diet induced obesity (DIO), Gal-3 was found to be increased in serum, white adipose tissue (WAT), and liver^9,10^. As compared to wild type mice, the Gal-3 gene knockout mice had reduced body weight and WAT, and were protected from DIO^11^.

Gal-3 stimulates the differentiation and proliferation of adipocytes. During the differentiation and maturation of adipocytes, the mRNA and protein expression levels of Gal-3 increased in a time-dependent manner^10,11^. Furthermore, knockout or knockdown of Gal-3 gene blocked the differentiation of adipocytes, as indicated by the reduction of cell size and lipid accumulation, as well as the down-regulation of key factors involved in adipogenesis, such as peroxisome proliferator-activated receptor γ (PPARγ) and CCAAT/enhancer-binding protein α (C/EBPα)^11^. In addition, the mRNA level of Gal-3 was up-regulated in growing but not quiescent adipocytes, and administration of exogenous Gal-3 protein promoted DNA synthesis and increased cell number of adipocytes *in vitro*^12^.

Berberine (BBR) is a multi-target natural product with a variety of pharmacological activities. Recent studies have proved that it has beneficial effects against various chronic diseases, which include metabolic disorders and obesity^13,14^. In mice with DIO, BBR suppressed extra body weight gain and reduced the weight of WATs^15,16^. In human subjects with non-alcoholic fatty liver disease (NAFLD) and obesity, BBR effectively reduced body weight, body mass index (BMI), and waist^17^. In accordance with the anti-obesity effect, BBR inhibited the differentiation and proliferation of 3T3-L1 adipocytes, as indicated by the reduction of lipid accumulation, the down-regulation of adipogenic genes, and the inhibition of DNA synthesis^1,18–21^.

The molecular mechanisms of BBR to suppress the differentiation and proliferation of adipocytes are not fully elucidated. In previous studies, transcription factors such as cAMP-response element-binding protein (CREB)^1^ and GATA binding proteins^18^ were shown to be involved in the differentiation-inhibitory effect of BBR. However, the detailed mechanisms still need further investigation. In the current study, we report that BBR down-regulates Gal-3, which is associated with its inhibitory effects on the differentiation and proliferation of adipocytes and its anti-obesity activity.

## Results

### BBR suppresses the differentiation of preadipocytes through down-regulating Gal-3

**Figure 1:**
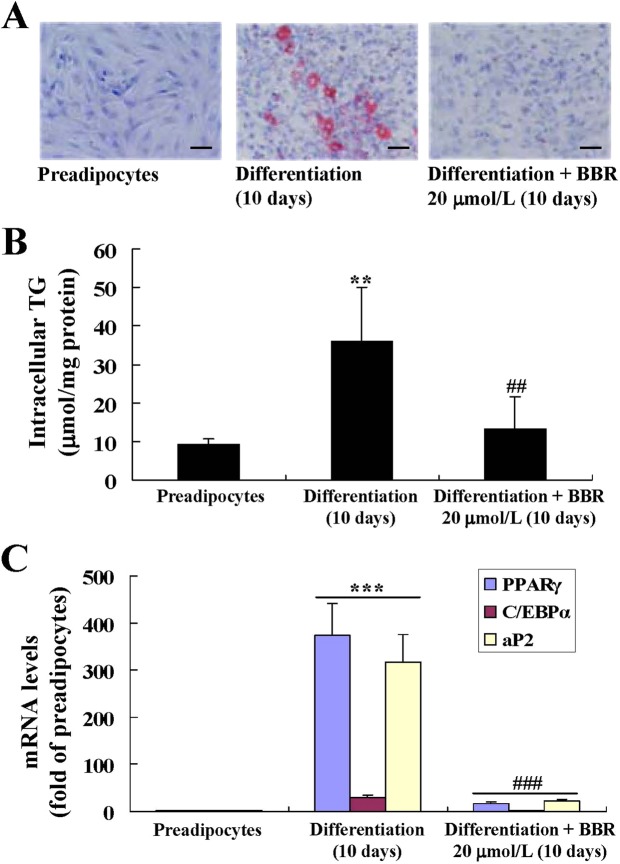
Effects of BBR on differentiation of mouse primary preadipocytes. Mouse primary preadipocytes were left untreated or induced for differentiation, during which the cells were left untreated or treated with BBR as indicated. After treatment, cells were subjected to oil red O (ORO) staining and photographed under a light microscope (×400, scale bar = 100 μm). (**A**) Intracellular triglyceride (TG) was determined and normalized to protein concentrations. (**B**) The mRNA expression levels of adipogenic genes were analyzed by real time RT-PCR, which were normalized to β-actin (ACTB) and presented as fold of preadipocytes. (**C**) Values are mean ± SD of 3–4 separate experiments. **p < 0.01, ***p < 0.001 *vs*. that of preadipocytes; ^##^*p* < 0.01, ^###^*p* < 0.001 *vs*. that of differentiated adipocytes left untreated.

Mouse primary preadipocytes differentiated into mature adipocytes after 10 days of induction (Fig. 1A–C). BBR at a concentration of 20 μmol/L suppressed the differentiation of preadipocytes almost completely, as indicated by the significantly reduction of lipid accumulation (Fig. 1A), the decrease of intracellular triglyceride (TG) (Fig. 1B), and the down-regulation of typical adipogenic genes (Fig. 1C) (*p* < 0.01 or *p* < 0.001 *vs*. differentiated adipocytes left untreated).

**Figure 2:**
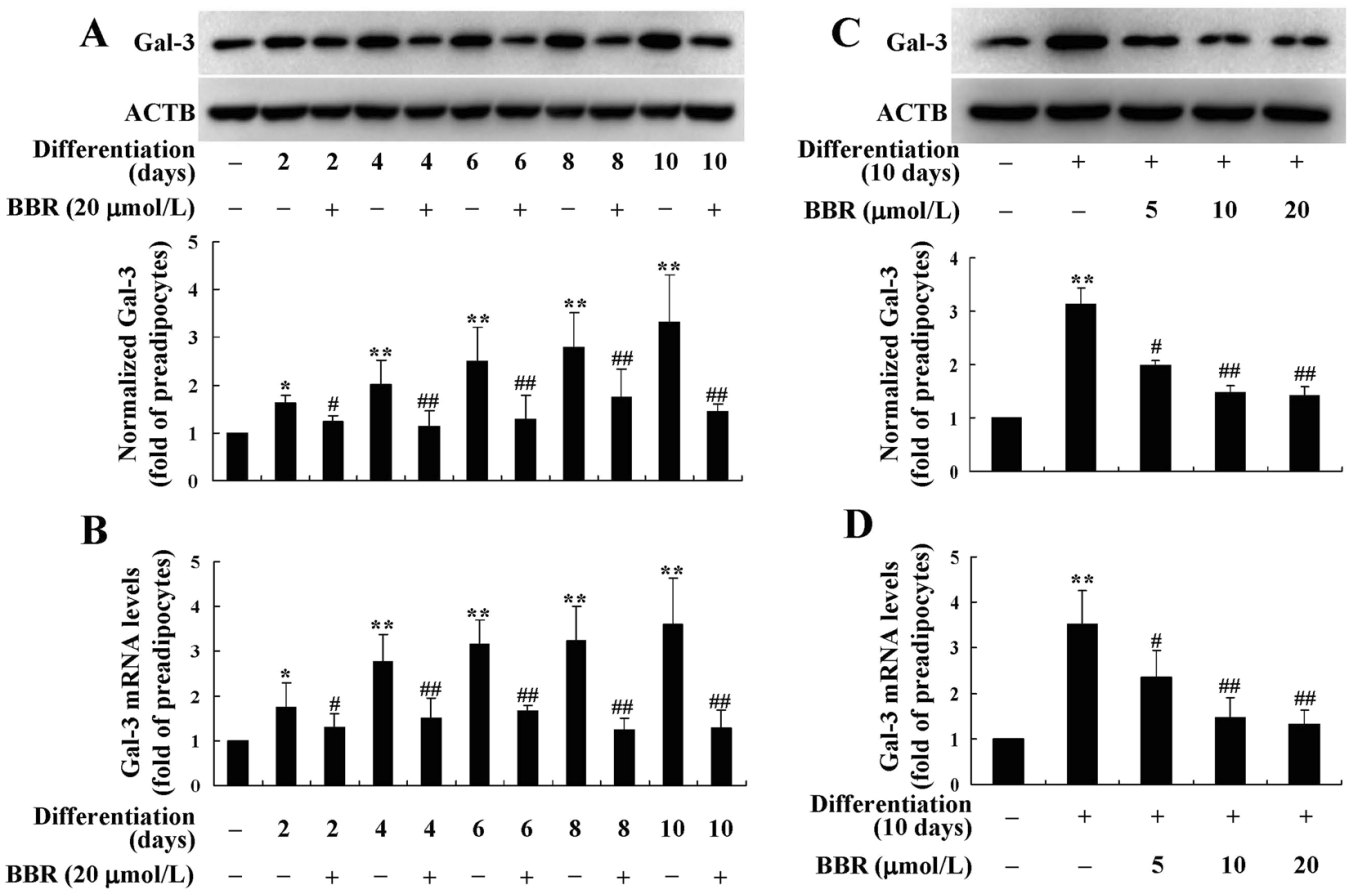
Effects of BBR on Gal-3 expression during differentiation of mouse primary preadipocytes. Mouse primary preadipocytes were left untreated or induced for differentiation. During induction, cells were left untreated or treated with BBR at 20 μmol/L for different time intervals, and cells were harvested for western blot (**A**) or real time RT-PCR (**B**) analysis of Gal-3 protein or mRNA levels. Alternatively, cells were left untreated or treated with different concentrations of BBR during the 10 days of differentiation period, and Gal-3 protein (**C**) and mRNA (**D**) levels were determined. The expression levels of Gal-3 were normalized to ACTB and presented as fold of preadipocytes. Values are mean ± SD of 3–4 separate experiments. *p < 0.05, **p < 0.01 *vs*. that of preadipocytes; ^#^*p* < 0.05, ^##^*p* < 0.01 *vs*^.^ that of differentiated adipocytes left untreated.

Accompanied with the differentiation of preadipocytes, the protein (Fig. 2A) and mRNA (Fig. 2B) expression levels of Gal-3 increased in a time-dependent manner (*p* < 0.05 or *p* < 0.01 *vs*. preadipocytes). Co-administration of BBR at a concentration of 20 μmol/L prevented the up-regulation of Gal-3 effectively, and the efficacies became obvious from the 2th day of preadipocyte differentiation (*p* < 0.05 *vs*. differentiated adipocytes left untreated). After 10 days of differentiation and treatment, BBR also decreased the protein (Fig. 2C) and mRNA (Fig. 2D) expression levels of Gal-3 in a concentration-dependent manner, and 5 μmol/L of BBR was able to cause a significant reduction of Gal-3 (*p* < 0.05 *vs*. differentiated adipocytes left untreated).

**Figure 3:**
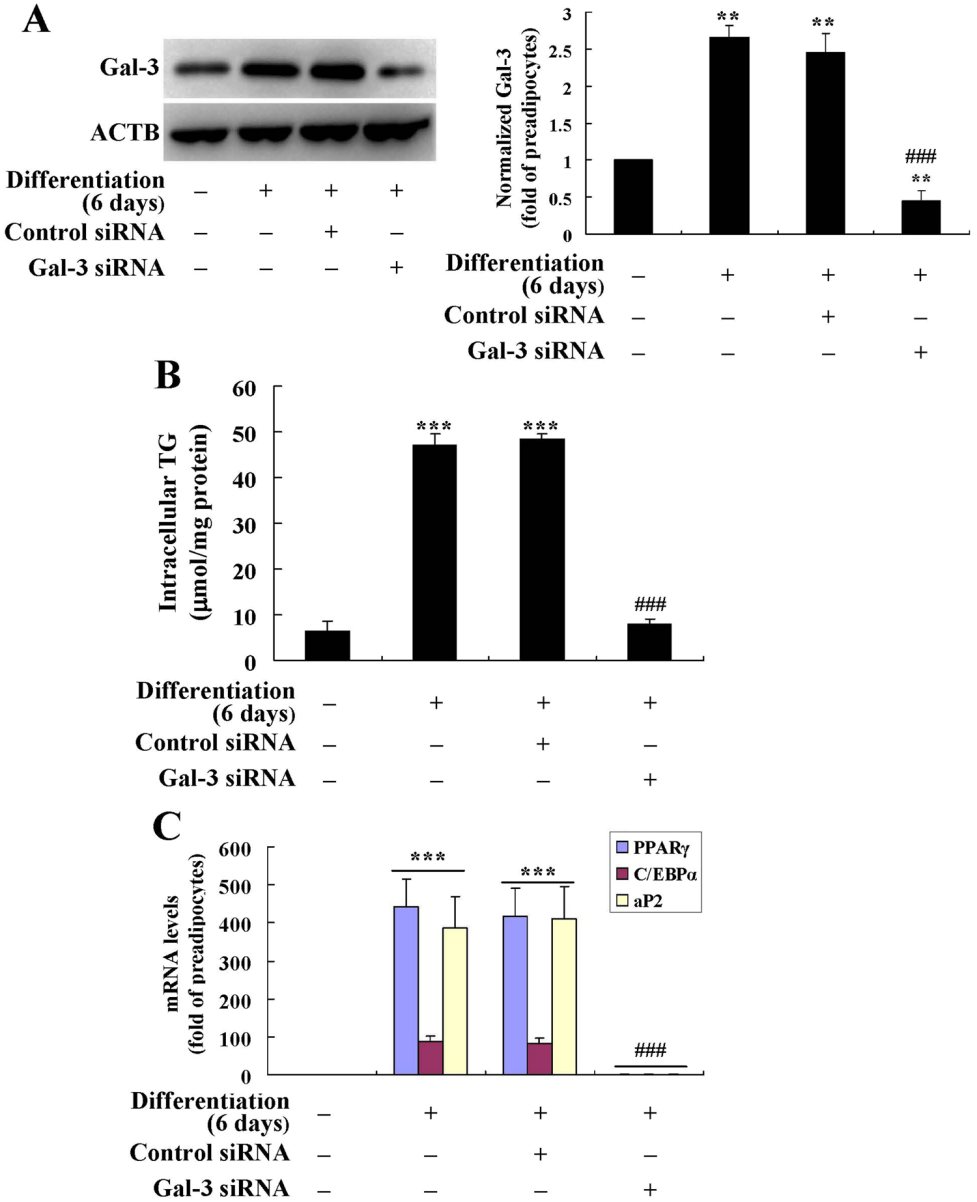
Effects of Gal-3 silencing on the differentiation of mouse primary preadipocytes. Mouse primary preadipocytes were transfected with Gal-3 or control siRNA and induced for differentiation for 6 days. Cells were harvested for western blot analysis of Gal-3 protein levels (**A**), intracellular TG (**B**) and the mRNA levels of adipogenic genes (**C**) were also determined. The protein or mRNA levels of target genes were normalized to ACTB and presented as fold of preadipocytes. Values are mean ± SD of 3–4 separate experiments. **p < 0.01, ***p < 0.001 *vs*. that of preadipocytes; ^###^*p* < 0.001 *vs*. that of differentiated adipocytes left untransfected or transfected with control siRNA.

For comparison, the effects of Gal-3 silencing on preadipocyte differentiation were determined. The results showed that as compared to differentiated adipocytes left untransfected or transfected with control siRNA (*p* < 0.001), the mouse Gal-3 siRNA greatly down-regulated Gal-3 protein expression (Fig. 3A) and suppressed the differentiation of mouse primary preadipocytes, as indicated by a significant reduction of intracellular TG (Fig. 3B) and adipogenic gene expression (Fig. 3C) after transfection.

**Figure 4:**
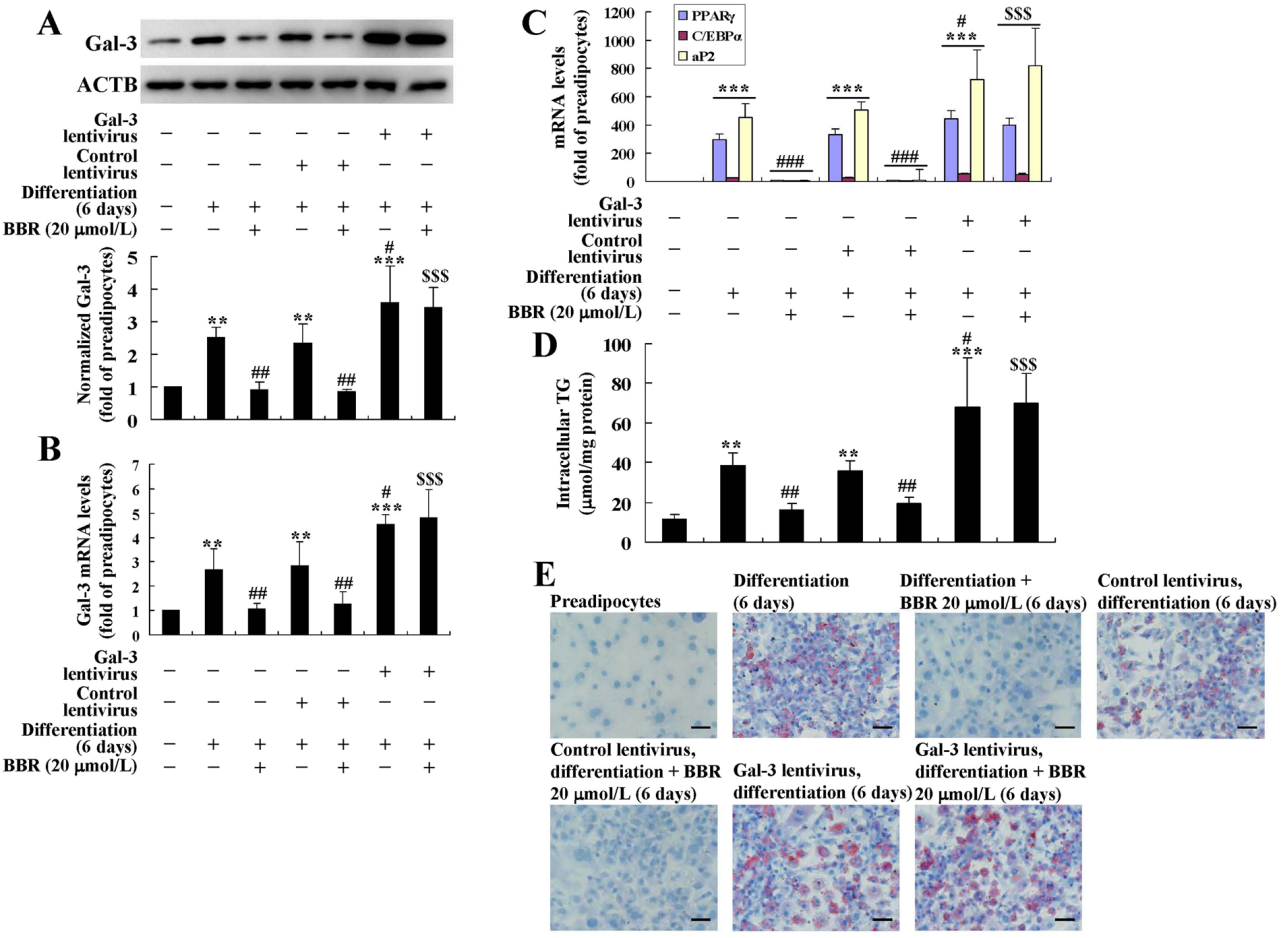
Effects of Gal-3 overexpression on the activities of BBR in differentiated mouse primary adipocytes. Mouse primary preadipocytes were left untreated or infected with Gal-3 or Control Lentiviral Activation Particles and were induced for differentiation for 6 days. During differentiation, the cells were left untreated or treated with BBR at 20 μmol/L. After treatment, cells were harvested for western blot and real-time RT-PCR analysis of Gal-3 protein (**A**) and mRNA levels (**B**), as well as the mRNA levels of adipogenic genes (**C**), which were normalized to ACTB and presented as fold of preadipocytes. In parallel experiments, cells were used for intracellular TG assay (**D**) and ORO staining (×400, scale bar = 100 μm). (**E**) Values are mean ± SD of 3–4 separate experiments. **p < 0.01, ***p < 0.001 *vs*. that of preadipocytes; ^#^*p* < 0.05, ^##^*p* < 0.01, ^###^*p* < 0.001 *vs*. that of differentiated adipocytes left uninfected or infected with control lentivirus and not treated with BBR; ^$$$^*p* < 0.001 *vs*. that of differentiated adipocytes left uninfected or infected with control lentivirus and treated with BBR.

In order to clarify the roles of Gal-3 in the activities of BBR to suppress preadipocyte differentiation, a lentiviral vector was used to infect the cells to induce Gal-3 overexpression. As shown in Fig. 4A,B, after infection of the Gal-3 Lentiviral Activation Particle and induction for differentiation for 6 days, the protein and mRNA expression levels of Gal-3 was significantly elevated as compared to differentiated adipocytes left uninfected or infected with control lentivirus (*p* < 0.05). In accordance with the successful induction of Gal-3 overexpression, the differentiation degree of adipocytes was elevated greatly (*p* < 0.05), as indicated by the further increases of adipogenic gene expression (Fig. 4C), intracellular TG (Fig. 4D), and lipid accumulation (Fig. 4E). The down-regulating effects of BBR on Gal-3 expression were blocked by the Gal-3 Lentiviral Activation Particle (Fig. 4A,B), and accordingly, the inhibitory effects on preadipocyte differentiation were completely abolished (Fig. 4C–E). These results prove that the down-regulation of Gal-3 by BBR is required for its activities to suppress preadipocyte differentiation.

### BBR suppresses the proliferation of preadipocytes through down-regulating Gal-3

**Figure 5:**
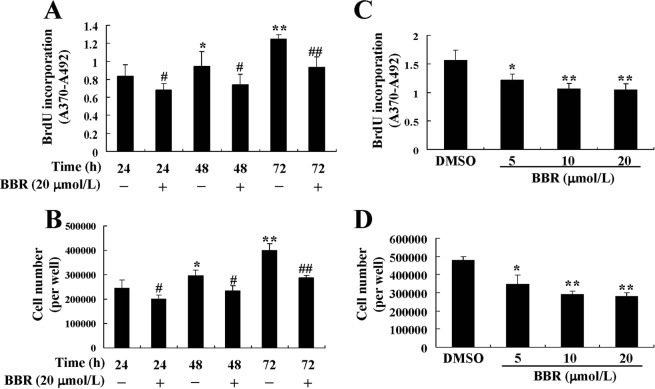
Effects of BBR on proliferation of mouse primary preadipocytes. Mouse primary preadipocytes were treated with DMSO or 20 μmol/L of BBR for different time intervals, BrdU was added at the same time and BrdU incorporation was assayed. (**A**) In a parallel experiment, cell numbers (**B**) were determined. Alternatively, cells were treated with DMSO or different concentrations of BBR for 72 h, and BrdU incorporation (**C**), and cell numbers (**D**) were determined. Values are mean ± SD of 4 separate experiments. (**A**,**B**) *p < 0.05, **p < 0.01 *vs*. that of cells treated with DMSO for 24 h; ^#^*p* < 0.05, ^##^*p* < 0.01 *vs*^.^ that of cells treated with DMSO for the same time interval. (C and D) *p < 0.05, **p < 0.01 *vs*. that of cells treated with DMSO.

5-bromo-2-deoxyuridine (BrdU) incorporation (Fig. 5A) and cell number (Fig. 5B) increased time-dependently during the culture of preadipocytes, which indicated a normal process of cell proliferation. However, in the presence of BBR at a concentration of 20 μmol/L, the proliferation of preadipocytes was totally inhibited (*p* < 0.05 or *p* < 0.01 *vs*. dimethyl sulfoxide [DMSO] treated cells). The inhibitory effects of BBR were also concentration-dependent, and 5 μmol/L of BBR treatment for 72 h was able to reduce BrdU incorporation (Fig. 5C) and cell number (Fig. 5D) significantly (*p* < 0.05 *vs*. DMSO treated cells). To rule out the possibility of cytotoxicity, lactate dehydrogenase (LDH) levels in culture supernatants were assayed, and BBR did not cause an increase of LDH release in this experiment (data not shown).

**Figure 6:**
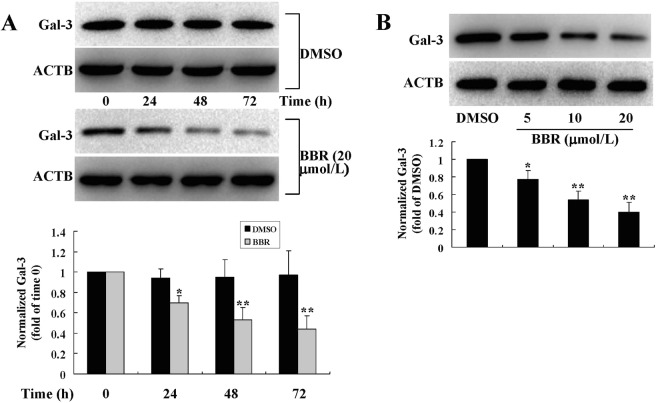
Effects of BBR on Gal-3 protein expression in mouse primary preadipocytes. Cells were treated with DMSO or BBR for different time intervals (**A**) or at different concentrations (**B**) as in Fig. 4. After treatment, cell total proteins were extracted for western blot analysis of Gal-3 expression, which was normalized to ACTB and presented as fold of control cells. Values are mean ± SD of 3 separate experiments. *p < 0.05, **p < 0.01 *vs*. that of cells treated with DMSO.

During the process of proliferation, the protein (Fig. 6) and mRNA (data not shown) expression levels of Gal-3 were significantly decreased by BBR treatment (*p* < 0.05 or *p* < 0.01 *vs*. DMSO treated cells), and the effects were both time-dependent (Fig. 6A) and concentration-dependent (Fig. 6B).

**Figure 7:**
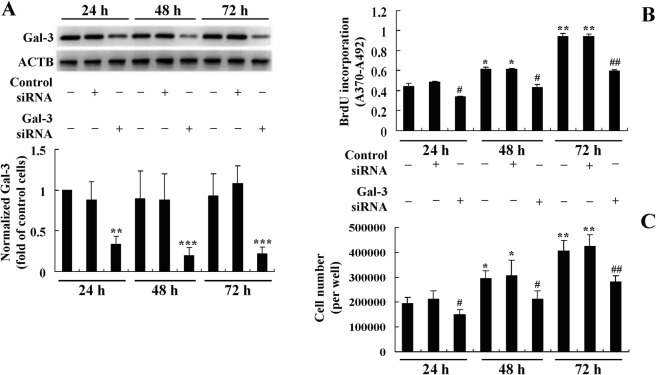
Effects of Gal-3 silencing on the proliferation of mouse primary preadipocytes. Mouse primary preadipocytes were transfected with Gal-3 or control siRNA, 24, 48, and 72 h after transfection, cells were harvested for western blot analysis of Gal-3 protein levels, which were normalized to ACTB and presented as fold of control cells (untransfected cells cultured for 24 h). (**A**) BrdU incorporation (**B**) and cell numbers (**C**) were also determined. Values are mean ± SD of 3–4 separate experiments. (**A**) **p < 0.01, ***p < 0.001 *vs*. that of untransfected or control siRNA transfected cells cultured for the same time interval. (**B**,**C**) *p < 0.05, **p < 0.01 *vs*. that of control cells; ^#^*p* < 0.05, ^##^*p* < 0.01 *vs*. that of untransfected or control siRNA transfected cells cultured for the same time interval.

For comparison, the effects of Gal-3 silencing on preadipocyte proliferation were determined. The results showed that as compared to untransfected or control siRNA transfected cells, the mouse Gal-3 siRNA greatly down-regulated Gal-3 protein expression (*p* < 0.01 or *p* < 0.001) (Fig. 7A) and suppressed the proliferation of mouse primary preadipocytes, as indicated by a significant reduction of BrdU incorporation (*p* < 0.05 or *p* < 0.01) (Fig. 7B) and cell number (*p* < 0.05 or *p* < 0.01) (Fig. 7C) after transfection.

**Figure 8:**
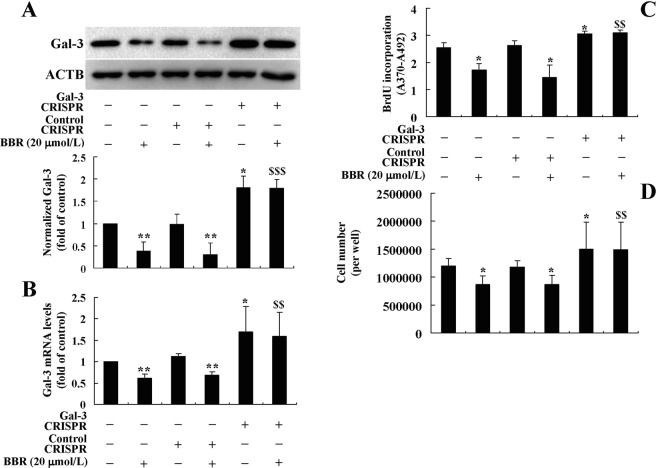
Effects of Gal-3 overexpression on the activities of BBR in mouse primary preadipocytes. Mouse primary preadipocytes were left untreated or transfected with Gal-3 or Control CRISPR Activation Plasmids as indicated. After 24 h of transfection, cells were left untreated or treated with 20 μmol/L of BBR for 48 h. After treatment, Gal-3 protein (**A**) and mRNA (**B**) levels were determined, which were normalized to ACTB and presented as fold of control cells. In parallel experiments, BrdU incorporation (**C**) and cell numbers (**D**) were determined. Values are mean ± SD of 3–4 separate experiments. *p < 0.05, **p < 0.01 *vs*. that of untransfected or control plasmid transfected cells not treated with BBR; ^$$^*p* < 0.01, ^$$$^*p* < 0.001 *vs*. that of untransfected or control plasmid transfected cells treated with BBR.

After transfection of the Gal-3 CRISPR Activation Plasmid, the protein (Fig. 8A) and mRNA (Fig. 8B) expression levels of Gal-3 increased significantly as compared to the untransfected or Control CRISPR Activation Plasmid transfected cells (*p* < 0.05). Accompanied with the overexpression of Gal-3, BrdU incorporation (Fig. 8C) and cell number (Fig. 8D) of preadipocytes increased greatly (*p* < 0.05). The Gal-3 CRISPR Activation Plasmid blocked the down-regulating effects of BBR on Gal-3 expression (Fig. 8A,B), and accordingly, totally abolished the inhibitory effects on preadipocyte proliferation (Fig. 8C,D). These results prove that in addition to inhibition of differentiation, the down-regulation of Gal-3 by BBR is also required for its activities to suppress preadipocyte proliferation.

### BBR down-regulates Gal-3 both transcriptionally and post-transcriptionally

**Figure 9:**
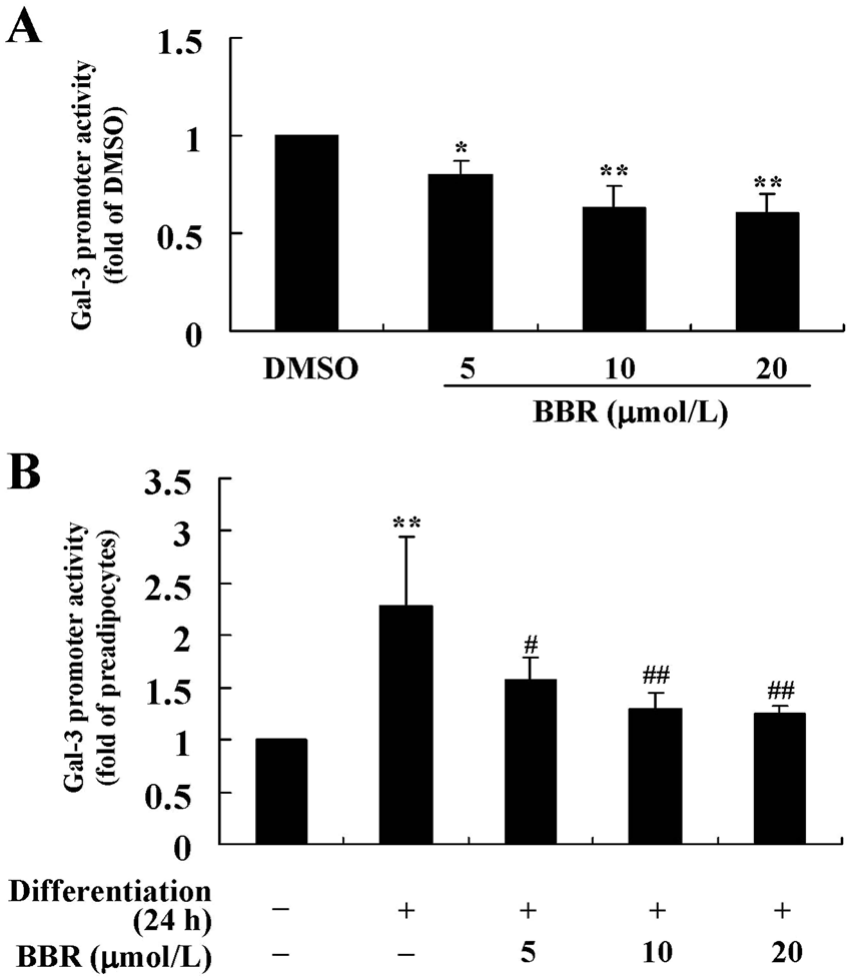
Effects of BBR on Gal-3 promoter activity. A recombinant plasmid expressing *luc2* reporter gene driven by the mouse Gal-3 promoter was co-tranfected with the pGL4.74[*hRluc*/TK] Vector into mouse primary preadipocytes. After 24 h of transfection, cells were treated with DMSO or different concentrations of BBR for 24 h. (**A**) Alternatively, cells were induced for differentiation with MDI for 24 h after transfection, and meanwhile DMSO or BBR were added and incubated. (**B**) After treatment, the activities of firefly luciferase and Renilla luciferase were determined. After normalization to Renilla luciferase, Gal-3 promoter activities were presented as fold of control cells. Values are mean ± SD of 4 separate experiments. (A) *p < 0.05, **p < 0.01 *vs*. that of DMSO. (B) **p < 0.01 *vs*. that of preadipocytes; ^#^*p* < 0.05, ^##^*p* < 0.01 *vs*. that of cells treated with MDI ^+^ DMSO.

After 24 h of treatment, BBR suppressed the promoter activity of Gal-3 gene in a concentration-dependent manner in preadipocytes (Fig. 9A), as indicated by a significant reduction of firefly luciferase activity (*p* < 0.05 or *p* < 0.01 *vs*. DMSO treated cells). After 24 h induction of differentiation, a significant increase of Gal-3 promoter activity was observed (*p* < 0.01 *vs*. preadipocytes), which was able to be prevented by co-administration of BBR (*p* < 0.05 or *p* < 0.01 *vs*. differentiated adipocytes treated with DMSO) (Fig. 9B).

**Figure 10:**
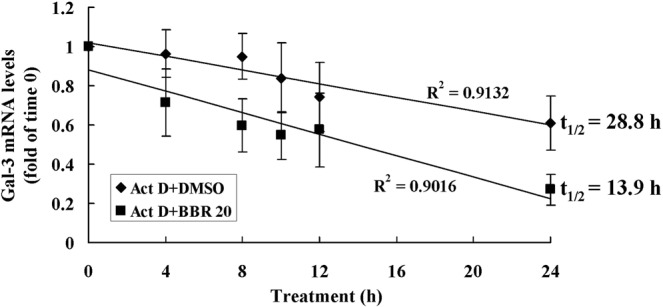
Effects of BBR on Gal-3 mRNA stability. Mouse primary preadipocytes were treated with 5 μg/mL of Act D together with DMSO or 20 μmol/L of BBR. Before treatment and at indicated time intervals after treatment, cells were harvested for real-time RT-PCR analysis of Gal-3 mRNA levels, which were normalized to ACTB and presented as fold of time 0, and values are mean ± SD of 4 separate experiments. The Gal-3 mRNAs were plotted against time and t_1/2_ values were calculated.

In addition to suppression of promoter activity, BBR also reduced the expression of Gal-3 at the post-transcriptional level through destabilizing its mRNA. As shown in Fig. 10, actinomycin D (Act D) was used to block mRNA synthesis of preadipocytes and Gal-3 mRNA stability was determined. When treated with DMSO, Gal-3 mRNA decayed slowly with a t_1/2_ value of about 28.8 h. However, BBR at a concentration of 20 μmol/L accelerated the decaying rate of Gal-3 mRNA greatly, and the t_1/2_ value was only about half of DMSO treated cells (Fig. 10). The destabilizing effect of BBR on Gal-3 mRNA was concentration-dependent, and 5 or 10 μmol/L of BBR was also able to cause an obvious acceleration of Gal-3 mRNA decay (data not shown).

**Figure 11:**
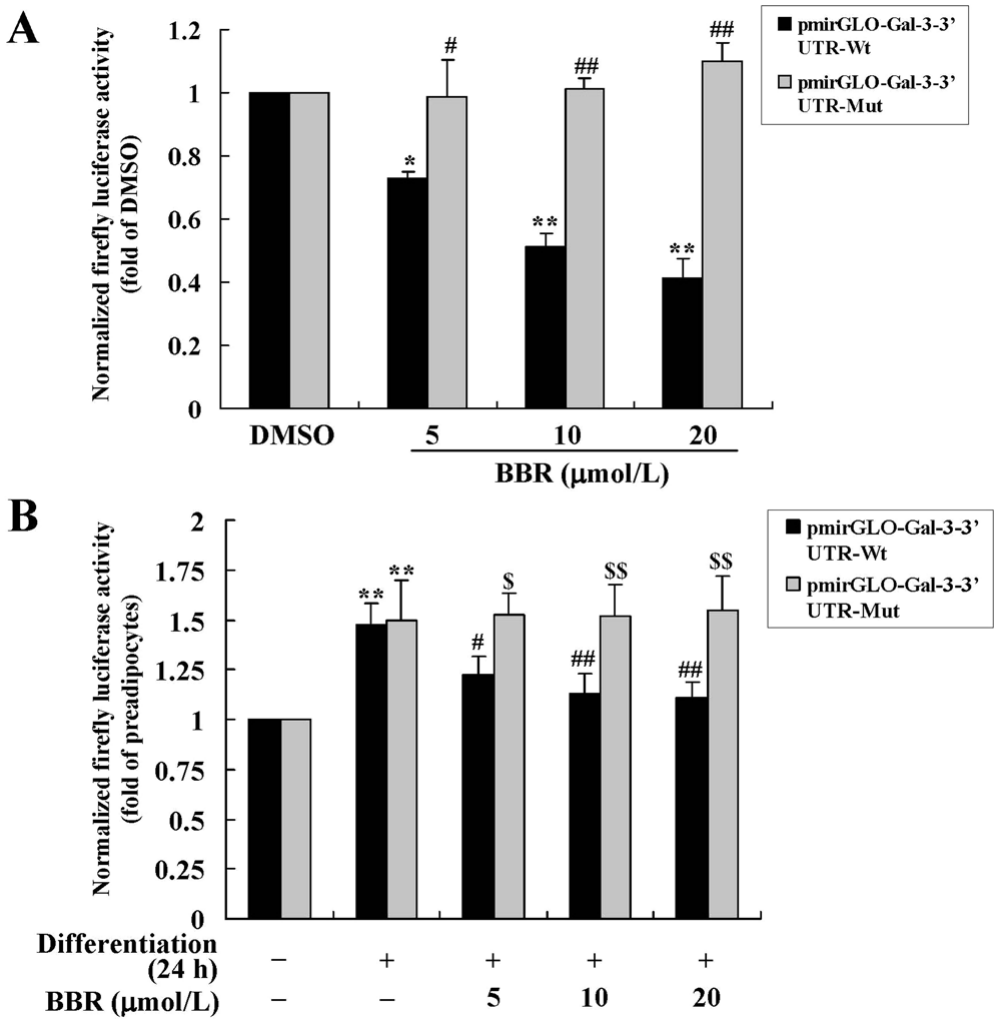
Effects of BBR on firefly luciferase activities of recombinant plasmids containing Gal-3 3′UTR. Recombinant dual-luciferase expressing plasmids containing wild type (Wt) or a point mutation (Mut) of mouse Gal-3 3′UTR were transfected into mouse primary preadipocytes. After 24 h of transfection, cells were treated with DMSO or different concentrations of BBR for 24 h. (**A**) Alternatively, cells were induced for differentiation with MDI for 24 h after transfection, and meanwhile DMSO or BBR were added and incubated. (**B**) After treatment, the activities of firefly luciferase were determined, normalized to Renilla luciferase activities, and presented as fold of control cells. Values are mean ± SD of 4 separate experiments. (**A**) *p < 0.05, **p < 0.01 *vs*. that of DMSO; ^#^*p* < 0.05, ^##^*p* < 0.01 *vs*. that of pmirGLO-Gal-3–3′UTR-Wt transfected cells. (**B**) **p < 0.01 *vs*. that of preadipocytes; ^#^*p* < 0.05, ^##^*p* < 0.01 *vs*. that of cells treated with MDI ^+^ DMSO; ^$^*p* < 0.05, ^$$^*p* < 0.01 *vs*. that of pmirGLO-Gal-3-3′UTR-Wt transfected cells.

In order to confirm the post-transcriptional regulation of Gal-3 expression by BBR, a recombinant plasmid containing the *luc2* reporter gene and mouse Gal-3 3′ untranslated region (UTR) (Wt) was used to transfect mouse primary preadipocytes. As shown in Fig. 11A, BBR treatment caused a concentration-dependent reduction of the firefly luciferase activity (*p* < 0.05 or *p* < 0.01 *vs*. DMSO treated cells). Twenty-four-hour induction of differentiation caused a significant increase of firefly luciferase activity (*p* < 0.01 *vs*. preadipocytes), and co-administration of BBR suppressed this effect (*p* < 0.05 or *p* < 0.01 *vs*. differentiated adipocytes treated with DMSO) (Fig. 11B). These results indicate that 3′UTR is involved in the activity of BBR to down-regulate Gal-3 expression.

**Figure 12:**
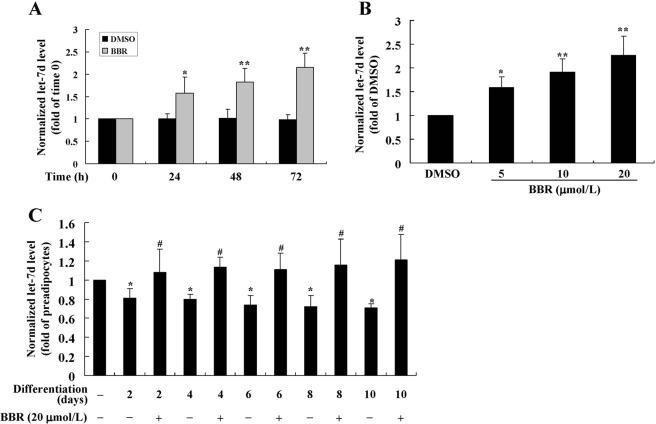
Effects of BBR on let-7d expression. Mouse primary preadipocytes were treated with DMSO, 20 μmol/L of BBR for different time intervals (**A**), or different concentrations of BBR for 72 h. (**B**) Alternatively, preadipocytes were left untreated or induced for differentiation. (**C**) During induction, cells were left untreated or treated with 20 μmol/L of BBR for different time intervals. After treatment, cells were harvested for real-time RT-PCR analysis of let-7d expression levels, which were normalized to RNU6-2 and presented as fold of control cells. Values are mean ± SD of 4 separate experiments. *p < 0.05, **p < 0.01 *vs*. that of control cells; ^#^*p* < 0.05 *vs*. that of differentiated adipocytes left untreated.

A number of microRNAs (miRNAs) such as let-7d^22^, miR-21^23^, miR-199a^24^, miR-322^25^ and miR-128^26^ are involved the post-transcriptional regulation of Gal-3. We determined the effects of BBR on their expression and found that it did not influence the levels of miR-21, miR-199a, miR-322 and miR-128 (Supplementary Fig. 1). In preadipocytes, BBR increased the expression level of let-7d in a time- (Fig. 12A) and concentration- (Fig. 12B) dependent manner (*p* < 0.05 or *p* < 0.01 *vs*. DMSO treated cells). During the process of differentiation (Fig. 12C), the expression level of let-7d declined significantly (*p* < 0.05 *vs*. preadipocytes), which was prevented by co-administration of BBR at a concentration of 20 μmol/L (*p* < 0.05 *vs*. differentiated adipocytes left untreated).

In order to validate the role of let-7d in the activity of BBR to modulate Gal-3 3′UTR, a point mutation was introduced in the let-7d binding site^22^ and the recombinant plasmid was used for transfection. The results showed that the inhibitory effects of BBR on firefly luciferase activity was totally abolished by the point mutation, either in preadipocytes (Fig. 11A) or in cells induced for differentiation (Fig. 11B) (*p* < 0.05 or *p* < 0.01 *vs*. pmirGLO-Gal-3-3′UTR-Wt transfected cells). These results prove that BBR down-regulates Gal-3 post-transcriptionally through let-7d-mediated modulation of 3′UTR.

### BBR down-regulates Gal-3 and inhibits adiposity in mice fed a high-fat diet (HFD)

The results showed that mice fed a HFD ate less as compared to those fed a normal diet (ND) (*p* < 0.01) (Table 1). However, the amount of food intake did not have statistical difference between the HFD-fed groups. Eight weeks of HFD-feeding caused a significant increase of body weight and body weight gain of the mice (*p* < 0.05 or *p* < 0.01 *vs*. ND group), which was effectively prevented by 100 mg/kg of BBR treatment (*p* < 0.05 or *p* < 0.01 *vs*. HFD group) (Table 1). HFD-feeding induced hyperlipidemia, hyperglycemia, hyperinsulinemia, and insulin resistance of the mice (*p* < 0.05, *p* < 0.01, or *p* < 0.001 *vs*. ND group). In accordance with the anti-obesity effect, BBR reduced serum lipids, glucose and insulin levels and ameliorated insulin resistance of the mice significantly (*p* < 0.05 or *p* < 0.01 *vs*. HFD group) (Table 1).

**Table 1:** Effetcs of BBR on body weight, metabolic parameters, WAT weight, eWAT TG, cell number, and serum Gal-3 in mice fed a HFD.

	ND	HFD	HFD + BBR
**Body weight (g)**			
Before	20.4 ± 1.56	20.4 ± 1.10	20.6 ± 1.23
After	27.2 ± 1.33	30.5 ± 3.09*	26.8 ± 3.31^#^
Body weight gain (g)	6.82 ± 0.35	10.1 ± 0.68**	6.17 ± 0.55^##^
Food intake (g/mouse/day)	4.45 ± 0.16	2.87 ± 0.17**	2.74 ± 0.19**
**Metabolic parameters**			
Serum CHO (mmol/L)	2.77 ± 0.54	4.32 ± 0.42**	3.41 ± 0.52^#^
Serum LDL-c (mmol/L)	0.34 ± 0.08	0.55 ± 0.14**	0.42 ± 0.09^#^
Serum TG (mmol/L)	0.70 ± 0.19	1.28 ± 0.29**	0.92 ± 0.22^#^
Serum Glucose (mmol/L)	5.20 ± 0.32	6.56 ± 0.95*	5.35 ± 0.58^#^
Serum Insulin (μU/mL)	3.44 ± 1.09	10.4 ± 2.10***	6.69 ± 1.82^#^
HOMA-IR	0.80 ± 0.14	3.03 ± 0.47***	1.59 ± 0.26^##^
**WAT weight (g)**			
Epididymal	0.18 ± 0.04	0.69 ± 0.12**	0.45 ± 0.10^#^
Perirenal	0.03 ± 0.01	0.39 ± 0.05***	0.26 ± 0.04^#^
Inguinal	0.09 ± 0.02	0.51 ± 0.06***	0.36 ± 0.05^#^
eWAT TG (μmol/mg protein)	1.10 ± 0.26	3.01 ± 0.42**	1.76 ± 0.33^#^
eWAT cell number (×10^6^/mouse)	3.85 ± 0.38	4.66 ± 0.55*	3.52 ± 0.49^#^
Serum Gal-3 (ng/mL)	16.1 ± 3.14	33.6 ± 6.06**	23.8 ± 4.63^#^

Values are mean ± SD of 10 mice in each group, except that eWAT cell numbers are mean ± SD of 5 mice in each group.

*p < 0.05, **p < 0.01, ***p < 0.001 *vs*. that of ND group; ^#^*p* < 0.05, ^##^*p* < 0.01 *vs*. that of HFD group.

**Figure 13:**
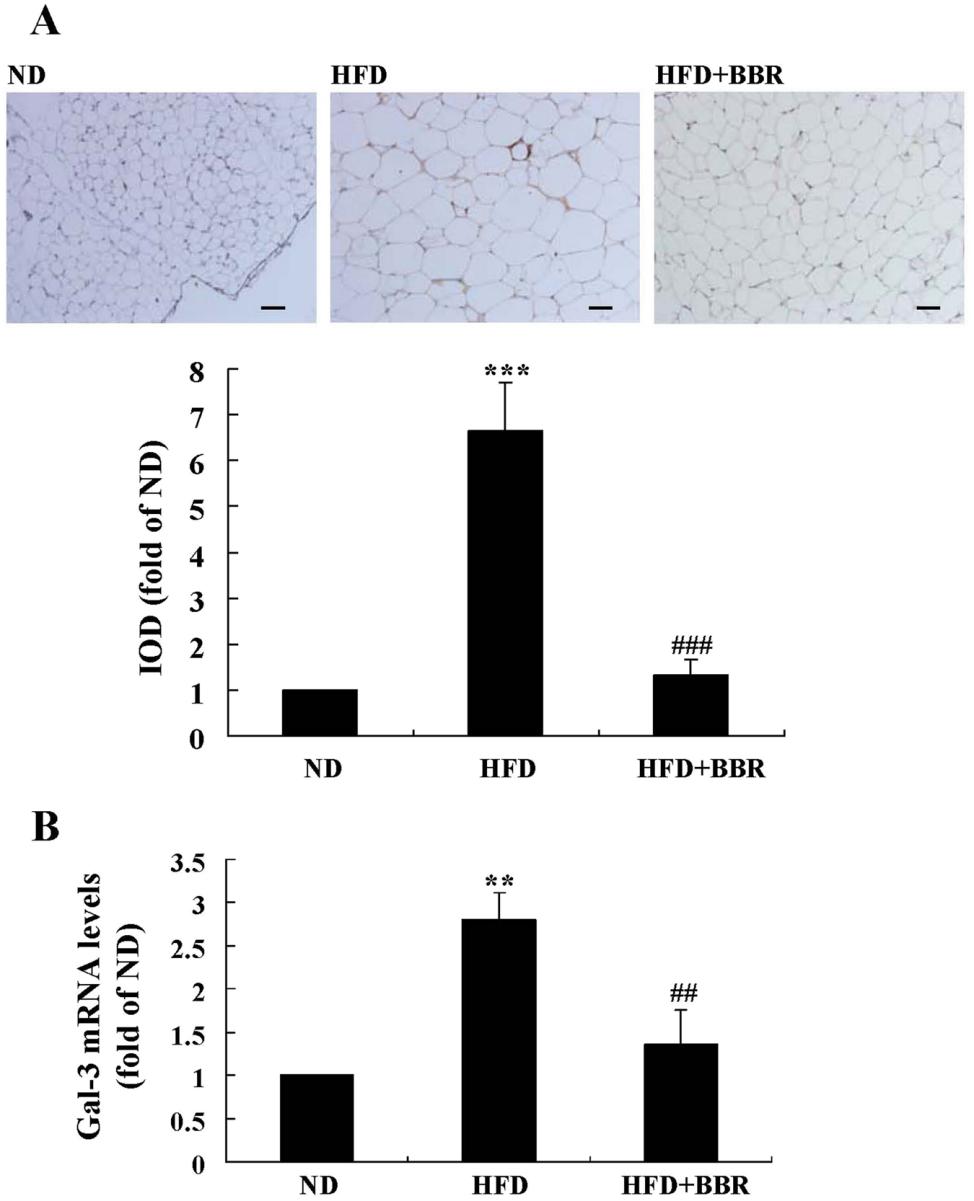
Effects of BBR on eWAT Gal-3 expression in mice fed a HFD. Male C57BL/6 J mice were grouped and treated as described in “Methods”. At the end of experiment, mice were sacrificed, and eWATs were subjected to paraffin sectioning. After dewaxing, Gal-3 protein expression levels were analyzed by IHC staining(×200, scale bar = 400 μm) (**A**, upper panel) and integrated optical density (IOD) values (**A**, lower panel) were determined. Meanwhile, Gal-3 mRNA expression levels in eWATs were analyzed by real-time RT-PCR, normalized to ACTB, and presented as fold of ND group (**B**). Values are mean ± SD of 10 mice in each group. **p < 0.01, ***p < 0.001 *vs*. that of ND group; ^##^*p* < 0.01, ^###^*p* < 0.001 *vs*. that of HFD group.

**Figure 14:**
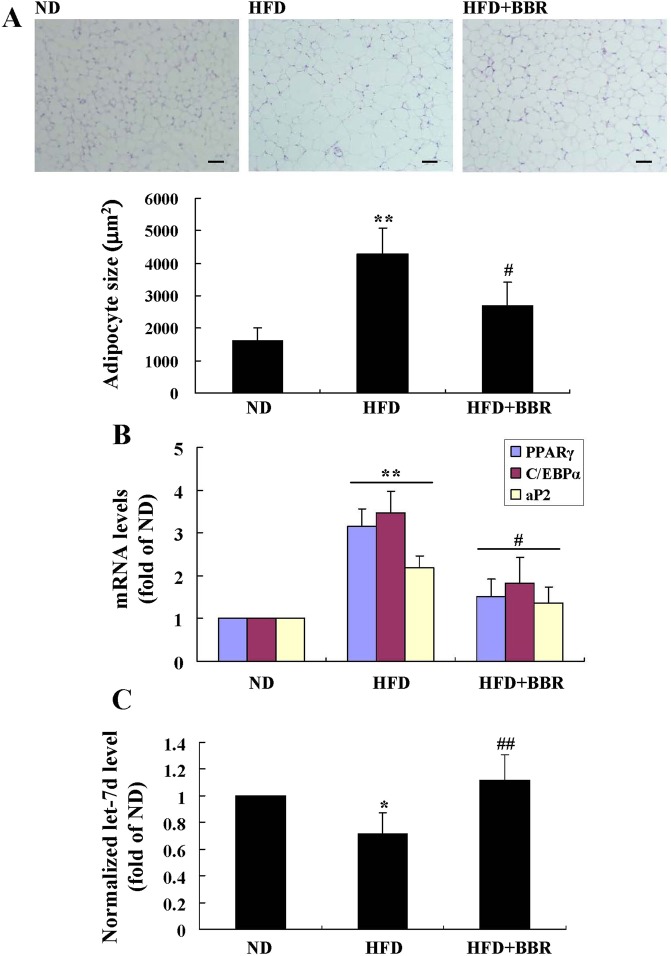
Effects of BBR on eWAT morphology and the levels of adipogenic genes and let-7d in mice fed a HFD. At the end of experiment, mice were sacrificed, their eWATs were harvested and subjected to paraffin sectioning and H&E staining (×200, scale bar = 400 μm) (**A**, upper panel), and adipocyte sizes were analyzed by the Image-Pro Plus 7 software (**A**, lower panel). The mRNA expression levels of adipogenic genes (**B**) and the levels of let-7d (**C**) in eWATs were analyzed by real-time RT-PCR and presented as fold of ND group. Values are mean ± SD of 10 mice in each group. *p < 0.05, **p < 0.01 *vs*. that of ND group; ^#^*p* < 0.05, ^##^*p* < 0.01 *vs*. that of HFD group.

**Figure 15:**
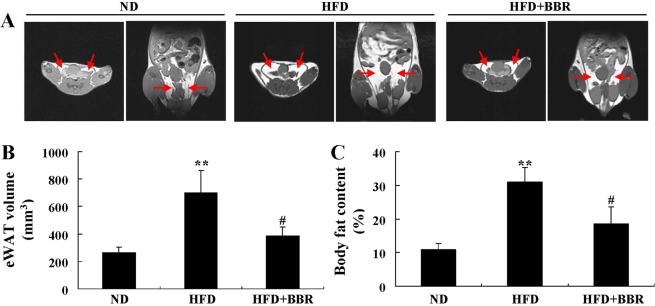
Effects of BBR on MRI performance in mice fed a HFD. Before the end of experiment, five mice in each group were subjected to MRI examination, during which axial (left panel of each group) and coronal (right panel of each group) scanning of eWATs (red arrows) were performed. (**A**) The volumes of eWATs (**B**) were analyzed by ImageJ. Body fat percentages (**C**) were calculated through analyzing fat peak and water peak. Values are mean ± SD of 5 mice in each group. **p < 0.01 *vs*. that of ND group, ^#^*p* < 0.05 *vs*. that of HFD group.

Serum Gal-3 level increased greatly in the HFD group as compared to ND group (*p* < 0.01) (Table 1). HFD-feeding induced an up-regulation of Gal-3 expression in mouse eWAT, as indicated by the significant increase of immunohistochemical (IHC) staining signal (Fig. 13A) as well as Gal-3 mRNA level (Fig. 13B) (*p* < 0.01 or *p* < 0.001 *vs*. ND group). Accordingly, the differentiation and proliferation of adipocytes in eWAT were enhanced by HFD-feeding, as indicated by the significant increase of cell size (Fig. 14A), the up-regulation of adipogenic genes (Fig. 14B), the elevation of TG content, and the increase of cell number (Table 1) (*p* < 0.05 or *p* < 0.01 *vs*. ND group). As a result, HFD-feeding enhanced adiposity of the mice, as indicated by the expansion of eWAT volume (Fig. 15A,B) and the increases of WAT weights (Table 1) and body fat percentage (Fig. 15C) (*p* < 0.01 or *p* < 0.001 *vs*. ND group). BBR treatment effectively down-regulated Gal-3, suppressed adipocyte differentiation and proliferation, and reduced adiposity of the mice (*p* < 0.05, *p* < 0.01 or *p* < 0.001 *vs*. HFD group) (Table 1, Figs 13–15). In addition, BBR greatly increased the expression level of let-7d in eWAT (*p* < 0.01 *vs*. HFD group), which was down-regulated by HFD-feeding (*p* < 0.05 *vs*. ND group) (Fig. 14C).

## Discussion

In the present study, we report that natural product BBR has suppressive activity on Gal-3 in adipocytes, which is associated with its anti-obesity effect.

The main function of WAT is fat storage and its expansion is crucial for the development of obesity^27^. The expansion of WAT includes both hypertrophy (results from cell differentiation) and hyperplasia (results from cell proliferation) of adipocytes^1^. Our results prove that BBR suppresses the differentiation and proliferation of preadipocytes isolated from eWAT through down-regulating Gal-3, which contributes to the anti-obesity effect. It seems that in different cell types, BBR has different effects on cell differentiation. For example, in certain cancer cells, BBR was shown to inhibit cell proliferation but promote cell differentiation^28^. BBR inhibits cell proliferation perhaps through influencing cell cycle or suppressing DNA synthesis^19,28^. The detailed mechanisms of BBR to modulate cell differentiation are not clear and need further study.

In addition to WAT, the expression of Gal-3 was shown to be elevated in brown adipose tissue (BAT) of HFD-fed mice or *ob*/*ob* mice, accompanied by increased intracellular lipid accumulation and cell size, which indicated an enhancement of cell differentiation^10^. BBR greatly reduced the cell size, lipid accumulation, and weight of BAT in *db*/*db* obese mice in a previous report^29^. Whether or not the effects of BBR on BAT are associated with Gal-3 down-regulation needs further investigation. Although BBR reduced adiposity of BAT, it promoted the thermogenic programme, which was able to stimulate energy expenditure and led to weight loss^29^.

Concerning the role of Gal-3 in adiposity, conflicting data exist. For example, Gal-3 gene knockout mice were shown to have reduced adiposity according to the study of Baek *et al*.^11^. However, other reports suggested that Gal-3 gene knockout mice had increased adiposity and diet induced obesity (DIO), perhaps due to amplified chronic inflammation^30,31^. Scientific explanations for these discrepancies are not available now, which need further investigation. Our results are in agreement with those of Baek *et al*., as down-regulation of Gal-3 by drug intervention inhibits adiposity and DIO in our experiments.

BBR down-regulates Gal-3 both transcriptionally and post-transcriptionally, as proved by the reduction of promoter activity and the acceleration of mRNA turnover. For the first time, we demonstrate that BBR suppresses Gal-3 post-transcriptionally through increasing let-7d expression. The critical role of let-7d in the activity of BBR is verified by the point mutation experiment, in which the inhibitory effect of BBR on Gal-3 3′UTR is abolished by change of a single nucleotide on the let-7d binding site. Upon binding to complementary sequences on 3′UTR, miRNAs are able to modulate gene expression through degradation of mRNA or inhibition of translation process^25^. Our results indicate that after treatment, BBR up-regulates let-7d and destabilizes Gal-3 mRNA.

In addition to gene overexpression experiments, the key roles of Gal-3 down-regulation in the suppressive activities of BBR on preadipocyte differentiation and proliferation were also verified by gene silencing experiments. As by only silencing Gal-3, we could reproduce the effects of BBR, which is a direct evidence of our conclusions.

The down-regulating effects of BBR or RNA interference on Gal-3 were observed both in preadipocytes and in differentiated adipocytes. In differentiated adipocytes, accompanied with the down-regulation of Gal-3, the expression levels of adipogenic genes like PPARγ were also reduced by BBR or Gal-3 silencing. In addition, overexpression of Gal-3 induced a further up-regulation of PPARγ and abolished the down-regulating effect of BBR on it. These results indicate that Gal-3 is possibly a regulator of PPARγ and is upstream of it. In agreement with our results, the study by Baek *et al*. proved that Gal-3 had a direct interaction with PPARγ and modulated its transcriptional activity^11^. Intriguingly, there were reports showed that in 3T3-L1 adipocytes, PPARγ was a target of both let-7d^32^ and miR-27a^21^, and BBR was able to up-regulate these miRNAs, as proved by our results and a previous study^21^. Whether or not miR-27a is involved in the modulation of Gal-3 is currently unclear. These data suggest that a complex network may exist for the integration and modulation of miRNAs, Gal-3, and adipogenesis.

In addition to adiposity, Gal-3 was also involved in the fibrosis and remodeling of adipose tissues. For example, rats fed a HFD had an increased Gal-3 expression and collagen content in abdominal adipose tissue^33^. BBR was reported to suppress the accumulation of extracellular matrix (ECM) in WAT of HFD-fed mice, and the effect was dependent on the activation of AMP-activated protein kinase (AMPK)^34^. Results from the current study indicate that the protective effect of BBR on adipose tissue fibrosis may also be related to the down-regulation of Gal-3.

In addition to adipose tissue, high expression of Gal-3 was related to myocardial injury in rats with DIO, as indicated by lipid accumulation, mitochondrial damage, and the increase of fatty acid β-oxidation in the heart^35^. Due to the down-regulating effect on Gal-3, BBR may have beneficial effects on myocardial injury induced by obesity, which merit further investigation.

Gal-3 has multifaceted biological functions and is associated with the pathogenesis or development of a number of chronic diseases or pathological conditions, such as insulin resistance^36^, diseases of the cardiovascular, respiratory, digestive, urinary systems or cancer^37,38^. Recently, Gal-3 was identified as a useful tool for the diagnosis or therapy of some chronic diseases, which may include heart failure, atherosclerosis, chronic obstructive pulmonary disease (COPD), non-alcoholic steatohepatitis (NASH), renal failure, organ fibrosis/remodeling, and some types of cancers^37,38^. Currently, a number of Gal-3 inhibitors or antagonists have been developed for the treatment of human diseases and some candidates have entered the stage of clinical trial^37,38^. Ongoing data will unravel the safety and effectiveness of Gal-3 as a target for the therapy of chronic diseases.

It is worthwhile to mention that Gal-3 is closely related to the development of fibrosis or remodeling of the heart, lung, liver, and kidney, as demonstrated by animal experiments and a few clinical studies^37–39^. Interestingly, BBR has ameliorative effects on fibrosis or remodeling of these organs, which may be caused by different factors or diseases^40–43^. Whether or not the anti-fibrotic activities of BBR are associated with the down-regulation of Gal-3 needs further investigation.

The roles of Gal-3 in diabetes mellitus and complications were controversial. On one hand, Gal-3 was able to bind advanced glycation end products (AGEs) and facilitated their clearance^5,6^; Gal-3 gene knockout mice showed deteriorated diabetic glomerular injury^44,45^. On the other hand, Gal-3 was shown to be up-regulated in animal models of diabetic nephropathy, which exhibited renal injury, fibrosis, and failure^37,38^. It is likely that during the course of diabetes, the deteriorative effect of Gal-3 exceeds its beneficial effect on AGEs due to high expression, which will accelerate the progression of diabetic nephropathy. It is possible that the ameliorative effects of BBR on diabetic complications may also be related to the modulation of Gal-3, which needs further study.

Numerous studies support that BBR is a multi-target drug with beneficial effects on a wide range of chronic diseases^13,14^. The molecular bases for the activities of BBR are not fully elucidated and are under extensive investigation due to the promising effectiveness and safety of this drug^13,14^. Results of the present study clarify the down-regulating effect of BBR on Gal-3 in adipose tissue, which is closely associated with its anti-obesity effect. In addition, a recent study proves that through AMPK activation and nuclear factor-κB (NF-κB) inhibition, BBR down-regulates Gal-3 in oxidized low-density lipoprotein (ox-LDL) treated macrophages, which may be associated with its anti-atherosclerosis effect^46^. These findings are in agreement with our study and imply that the inhibitory effects of BBR on Gal-3 may be associated with its efficacies on other chronic diseases. It is of scientific significance to investigate the influences of BBR on Gal-3 when it is used to treat or prevent other chronic diseases, and it can be expected that the results may help to further understand the complex network of BBR’s drug activity.

In conclusion, our results demonstrate that BBR suppresses the differentiation and proliferation of adipocytes and reduces adiposity through down-regulating Gal-3. Our study unravels a new mechanism for the pharmacological activities of BBR and will provide new scientific evidence for the clinical application of this promising drug.

## Methods

### Reagents and kits

BBR, DMSO, dexamethasone, methylisobutylxanthine (IBMX), and the LDH Activity Assay Kit were purchased from Sigma-Aldrich Co., LLC. (St. Louis, MO, USA). The recombinant human insulin injection was purchased from Lilly France (Paris, France), the Steatosis Colorimetric Assay Kit was purchased from Cayman Chemical (Ann Arbor, MI, USA), and the Tissue TG Assay Kit was purchased from Applygen Technologies Inc. (Beijing, China). DMEM/F12(1:1), DMEM, fetal bovine serum (FBS), Opti-MEM™/Reduced Serum Medium, and other reagents required for cell culture were purchased from Gibco-Invitrogen (NY, USA). Reagents required for cell or tissue total RNA extraction, reverse transcription (RT), and quantitative real-time PCR were purchased from Qiagen (Hilden, Germany) or Applied Biosystems (Foster City, CA, USA). The rabbit polyclonal or monoclonal antibodies against Gal-3 (#12733) and β-actin (ACTB) (#4970), and a horseradish peroxidase (HRP)-linked goat anti-rabbit IgG (#7074) were purchased from Cell Signaling Technology, Inc. (Danvers, MA, USA). Reagents required cell or tissue protein extraction, quantification, electrophoresis, and western blot were purchased from Thermo Fisher Scientific Inc. (Waltham, MA, USA) or Millipore (Bille-rica, MA, USA). The mouse Gal-3 siRNA, control siRNA, siRNA transfection reagent, and siRNA transfection medium were purchased from Santa Cruz Biotechnology, Inc. (Dallas, TX, USA).

The Gal-3 and Control Lentiviral Activation Particles for mouse cells, and the reagents or antibiotics for infection or selection were purchased from Santa Cruz Biotechnology, Inc.. The Cell Proliferation ELISA, BrdU (colorimetric) Kit was purchased from Roche Applied Science (Indianapolis, IN, USA). The Gal-3 and Control CRISPR Activation Plasmids for mouse cells, UltraCruz^®^ Transfection Reagent, and Plasmid Transfection Medium were purchased from Santa Cruz Biotechnology, Inc.. The pGL4.10[*luc2*] Vector, pGL4.74[*hRluc*/TK] Vector, pmirGLO Dual-Luciferase miRNA Target Expression Vector, FuGENE^®^ HD Transfection Reagent, and Dual-Glo^®^ Luciferase Assay System were purchased from Promega (Beijing) Biotech Co., Ltd. (Beijing, China). Act D was purchased from Tocris Bioscience (Shanghai, China). The miScript Primer Assays for mouse miRNAs let-7d, miR-21, miR-199a, miR-322, miR-128, and RNU6-2, the miRNeasy Mini Kit, miScript II RT Kit, and miScript SYBR Green PCR Kit were purchased from Qiagen. The Gal-3 (Mouse) ELISA Kit was purchased from Aviscera Bioscience, Inc. (Santa Clara, CA, USA); reagents for determination of serum cholesterol (CHO), LDL cholesterol (LDL-c), TG, and glucose were purchased from Beijing Strong Biotechnologies, Inc. (Beijing, China); the Mouse Ultrasensitive Insulin ELISA Kit was purchased from ALPCO (Salem, OR, USA).

### Isolation and culture of mouse primary preadipocytes

The mouse epididymal white adipose tissue (eWAT) was isolated under a sterile condition and sliced into small aliquots with a volume about 1 mm^3^ each. The type I collagenase (Thermo Fisher Scientific Inc.) at a concentration of 1 mg/mL was used to treat the tissues. After 90 min of incubation at 37 °C, the samples were treated with a 70 μm cell filter and the filtrates were centrifuged at 1200 rpm for 8 min. The precipitates were resuspended in DMEM/F12(1:1) and were centrifuged at 1200 rpm for 8 min again. The cells were then cultured in DMEM/F12(1:1) + 10% FBS at 37 °C in an atmosphere of 5% CO_2_. For the induction of differentiation, DMEM containing 10% FBS plus 0.5 mmol/L of IBMX, 1 μmol/L of dexamethasone, and 5 μg/mL of insulin (MDI) was used.

### Cell oil red O (ORO) staining and TG assay

Preadipocytes seeded onto 6-well plates were left untreated or subjected to differentiation and appropriate treatment. A Steatosis Colorimetric Assay Kit was used for ORO staining according to the supplier’s protocol. Meanwhile, intracellular TG was determined by a kit and normalized to protein concentrations.

### RNA extraction and real-time RT-PCR

After treatment, total RNAs were extracted from cells or tissues and reversely transcribed into cDNAs as described before^47^. The cDNAs were used as templates in quantitative real-time PCR. The reactions were performed with gene specific primers (Supplementary Table 1) in an ABI 7500 Real-Time PCR system (Applied Biosystems). The reaction condition was 95 °C, 2 min, followed by 40 cycles of 95 °C, 15 sec, and 60 °C, 1 min. The comparative threshold cycle (C_T_) method was used for relative quantification of target genes, and ACTB was used as an internal control.

### Western blot

After treatment, cells and tissues were lysed or homogenized, and total proteins were extracted as described before^47^. The samples were quantified for protein concentration, and about 20 μg was subjected to 10% sodium dodecyl sulfate polyacrylamide gel electrophoresis (SDS-PAGE). The gels were electrically transferred to PVDF membranes, which were blocked and incubated with primary and secondary antibodies as described before^47^. After washing, the signals were developed with an ECL kit and quantified, with ACTB as an internal control.

### BrdU incorporation, cell counting, and LDH assay

Mouse primary preadipocytes were seeded onto 96-well plates and cultured for 24 h. Then, the BrdU labeling reagent was added and cells were treated as indicated. After treatment, the incorporation of BrdU was determined according to supplier’s protocol, which was based on ELISA. After color development, the absorbance at 370 nm was read by a microplate reader. The absorbance at 492 nm was used as a reference, and BrdU incorporation was presented as A370-A492^12^.

In a parallel experiment, mouse primary preadipocytes were seeded onto 6-well plates and cultured for 24 h. After treatment, cells were trypsinized and centrifuged at 1000 rpm for 5 min. The cells were then resuspended in phosphate buffer saline (PBS) and a blood cell counting chamber was used to count cells. Meanwhile, culture media were harvested after treatment. After centrifugation at 1000 rpm for 5 min, the supernatants were used for LDH activity assay by a commercial kit.

### siRNA transfection

For differentiation-induction experiments, mouse primary preadipocytes seeded onto 6-well plates were left untreated or transfected with Gal-3 or control siRNA for 7 h according to the supplier’s protocol. After transfection, cells were again left untreated or induced for differentiation for 6 days, and on the 3rd day of induction, the above siRNAs were used to transfect corresponding cells again. Then, Gal-3 protein levels, intracellular TG, and the mRNA levels of adipogenic genes were determined.

For proliferation experiments, preadipocytes were left untreated or transfected with Gal-3 or control siRNA for 7 h, at 24, 48, and 72 h after transfection, cells were harvested for western blot analysis of Gal-3 protein levels, BrdU incorporation assay, and cell counting.

### Overexpression of mouse Gal-3 gene

Mouse primary preadipocytes were seeded onto 12-well plates and cultured for 24 h. The cells were infected with Gal-3 or Control Lentiviral Activation Particles according to supplier’s protocol. Cells with stable infection were selected by antibiotic resistance to hygromycin B, blasticidin S, and zeocin. The stably infected cells were amplified and induced for differentiation as described earlier. At the same time of induction, the cells were left untreated or treated with BBR at a concentration of 20 μmol/L for 6 days. The protein and mRNA expression levels of Gal-3 and the mRNA levels of adipogenic genes were determined by western blot or real-time RT-PCR. In parallel experiments, cells were subjected to ORO staining and TG assay after treatment.

In another experiment, preadipocytes were transfected with Gal-3 or Control CRISPR Activation Plasmids, respectively. The plasmids were mixed with the UltraCruz^®^ Transfection Reagent and the Plasmid Transfection Medium according to supplier’s protocol. After 24 h of incubation, culture media were discarded and fresh media were replaced, the cells were left untreated or treated with BBR at a concentration of 20 μmol/L for 48 h. After treatment, the protein and mRNA expression levels of Gal-3, BrdU incorporation and cell numbers were determined.

### Plasmid construction, transient transfection, and luciferase assay

The promoter region (-1251- + 141) of mouse Gal-3 gene was amplified by PCR. The forward primer (5′ to 3′) is CGGGGTACCGGATCAAAGTTAGGCGTCGG, which contains a *Kpn*I restriction site (underlined), and the reverse primer (5′ to 3′) is CCGCTCGAGACCTCCGTCCATCTCCCG, which contains an *Xho*I restriction site (underlined). After purification, the fragment was properly digested and inserted into the multiple cloning region of pGL4.10[*luc2*] Vector, which was immediately upstream of the *luc2* reporter gene (Supplementary Fig. 2A). The recombinant Gal-3-pGL4.10[*luc2*] plasmid was selected and amplified, and the correct insertion of Gal-3 gene promoter was verified by DNA sequencing (BGI Shenzhen, Shenzhen, China).

A DNA fragment containing the 3′UTR (11822–12308 nt) of mouse Gal-3 gene (Gal-3-3′UTR-Wt) was obtained by chemical synthesis (BGI Shenzhen). In another fragment, a point mutation (C → G)^22^ was introduced in the let-7d binding site (11895–11916) of mouse Gal-3 3′UTR (Gal-3-3′UTR-Mut) (Supplementary Fig. 2B). There was a *Nhe*I restriction site at the 5′ end and an *Xho*I restriction site at the 3′ end of the fragments. After proper digestion, the fragments were inserted into the multiple cloning region of pmirGLO Dual-Luciferase miRNA Target Expression Vector, which was immediately downstream of the *luc2* reporter gene (Supplementary Fig. 2B). The recombinant plasmids were named as pmirGLO-Gal-3-3′UTR-Wt and pmirGLO-Gal-3-3′UTR-Mut, respectively, and the correct insertions of fragments were verified by DNA sequencing (BGI Shenzhen).

Mouse primary preadipocytes were seeded onto 6-well plates and cultured for 24 h in DMEM/F12(1:1) + 10% FBS free of antibiotics. For one well of the plate, 1 μg of the Gal-3-pGL4.10[*luc2*] plasmid was co-transfected with 1 μg of the pGL4.74[*hRluc*/TK] Vector, which was used as an internal control. For pmirGLO-Gal-3-3′UTR-Wt and pmirGLO-Gal-3-3′UTR-Mut, 1 μg of the plasmids was used to transfect one well of the plate, respectively. The above plasmids were mixed with the FuGENE^®^ HD Transfection Reagent and Opti-MEM™/Reduced Serum Medium according to supplier’s protocols.

After 24 h of incubation, the cells were treated with DMSO or BBR as indicated for 24 h. In parallel experiments, cells were induced for differentiation for 24 h, and at the same time of induction, DMSO or BBR were added and incubated. After treatment, cells were harvested and lysed; the activities of firefly luciferase and Renilla luciferase were determined by the Dual-Glo^®^ Luciferase Assay System in a luminometer, and the firefly luciferase activities were normalized to those of Renilla luciferase.

### Gal-3 mRNA stability assay

Mouse primary preadipocytes were seeded onto 6-well plates and cultured for 24 h. Act D at a concentration of 5 μg/mL was used to treat the cells. At the same time, cells were treated with DMSO or BBR at a concentration of 20 μmol/L. Before treatment and at 4, 8, 10, 12, and 24 h after treatment, cells were harvested; total RNAs were extracted for real-time RT-PCR analysis of Gal-3 mRNA levels. The remaining mRNAs were plotted against time and linear regression analysis was performed. R^2^ values were presented, and the half-life (t_1/2_) values of Gal-3 mRNA were calculated according to the formulas of linear regression.

### miRNA analysis

After treatment, cells were harvested, cell total RNAs containing miRNAs were extracted by the miRNeasy Mini Kit and subjected to real-time RT-PCR by the miScript II RT Kit and the miScript SYBR Green PCR Kit according to supplier’s protocols. RNU6-2 was used as an internal control miRNA. After normalization, the expression levels of target miRNAs were presented as fold of control cells.

### Animal experiments

The protocols of the animal experiments were reviewed and approved by the Research Committee of the Institute of Materia Medica, and animals were cared humanely according to the institutional guidelines of Chinese Academy of Medical Sciences (CAMS) & Peking Union Medical College (PUMC).

Male C57BL/6 J mice, weighing about 19.5 ± 1.10 g, were purchased from Beijing Vital River Laboratory Animal Technology Co., Ltd. (Beijing, China). The mice were housed in an air-conditioned room with regular light/dark cycle as described before^47^.

In the animal experiment, 30 mice were randomly divided into 3 groups, with 10 mice each. They were the ND group, HFD (Research Diets, Inc., New Brunswick, NJ, USA) group, and HFD + BBR group, respectively. The dose of BBR was 100 mg/kg, and it was orally administered to the mice at the same time of HFD-feeding for 8 weeks. Body weight and food intake of the mice were recorded 2 times a week.

Two days before termination, five mice were randomly selected form each group. After overnight fasting, they were induced for anesthesia with 3% isoflurane and maintained with 1.5% isoflurane in a Matrx VIP 3000 Anesthesia Machine (Matrix Medical Inc, Minneapolis, MN, USA). These mice were then subjected to magnetic resonance imaging (MRI) analysis using a PharmaScan 70/16US (Bruker, Ettlingen, Germany). Axial and coronal scanning of eWATs were performed using the T1_RARE (rapid acquisition with relaxation enhancement) sequence with the following parameters: TR/TE = 1500/7 ms, image size = 256 × 256, field of view (FOV) = 45 × 45 mm, averages = 5, slice thickness = 0.7 mm. The ImageJ software (National Institutes of Health, Bethesda, MD, USA) was used to calculate the volumes of eWATs. Fat peak and water peak of the mice were scanned using the Singlepulse_1H sequence with the following parameters: TR = 1000 ms, averages = 1, repetitions = 10, acquisition bandwidth = 200000 Hz, acquisition points = 300. Body fat percentages were calculated as described before^17^.

Before the last day of experiment, the mice were fasted overnight, blood samples were taken by eyeball removal, and serums were isolated for the determination of CHO, LDL-c, TG, glucose, insulin, and Gal-3 levels using commercially available kits. The values of homeostasis model assessment-insulin resistance (HOMA-IR) were calculated as described before^48^. The mice were sacrificed; epididymal, perirenal, and inguinal WATs were isolated and weighed. A portion of eWAT from the same site was immediately fixed in 4% formaldehyde for paraffin embedding, sectioning, and hematoxylin and eosin (H&E) staining. After photographing, the Image-Pro Plus 7 software (Media Cybernetics, Inc., Rockville, MD, USA) was used to measure the sizes of adipocytes by analyzing 4 fields from each section. The diameters of adipocytes were also measured, which were used for the calculation of cell volumes. The cell numbers of eWATs were calculated as eWAT volume/cell volume.

After dewaxing, the sections were subjected to IHC staining for Gal-3 protein expression as described before^49^. After photographing, the Image-Pro Plus 7 software was used for the analysis of integrated optical density (IOD) values.

The rest of eWAT was quickly frozen in liquid nitrogen. TG contents of eWATs were determined and normalized to protein concentrations. The mRNA expression levels of Gal-3 and adipogenic genes were determined by real-time RT-PCR. In addition, the expression levels of let-7d in mouse eWATs were also measured.

### Statistical analysis

For *in vitro* studies, values are presented as mean ± SD of 3-4 separate experiments. For *in vivo* studies, values are mean ± SD of 10 or 5 (for MRI data and eWAT cell number) mice in each group. The GraphPad Prism 6 software (GraphPad Software Inc., La Jolla, CA, USA) was used for statistical analysis. After validation of the test for homogeneity of variance, differences among groups were examined by one-way ANOVA followed by Newman-Keuls (NK) test for multiple comparisons, *p* < 0.05 was considered to be statistically different.

## Supplementary information

Supplementary information
