# Supplementary material for: Berberine inhibits adipocyte differentiation, proliferation and adiposity through down-regulating galectin-3
Source: Sci Rep. 2019 Sep 16;9:13415. doi: 10.1038/s41598-019-50103-5 (PMC6746795; doi:10.1038/s41598-019-50103-5)

Supplementary Information

Berberine inhibits adipocyte differentiation, proliferation and adiposity through down-regulating galectin-3

Can Wang[1], Yan Wang[2], Shu-Rong Ma[2], Zeng-Yan Zuo[3], Yan-Bin Wu[3], Wei-Jia Kong[3], Ai-Ping Wang[1] & Jian-Dong Jiang[2]

[1]New Drug Safety Evaluation Center, Institute of Materia Medica, Chinese Academy of Medical Sciences and Peking Union Medical College, Beijing, China. [2]State Key Laboratory of Bioactive Natural Products and Function, Institute of Materia Medica, Chinese Academy of Medical Sciences and Peking Union Medical College, Beijing, China. [3]Department of Virology & NHC Key Laboratory of Biotechnology of Antibiotics, Institute of Medicinal Biotechnology, Chinese Academy of Medical Sciences and Peking Union Medical College, Beijing, China.

Correspondence and requests for materials should be addressed to W.J.K. (email: wjkong894@163.com), A.P.W. (email: wangaiping@imm.ac.cn), and J.D.J. (email: jiangjd@imm.ac.cn).

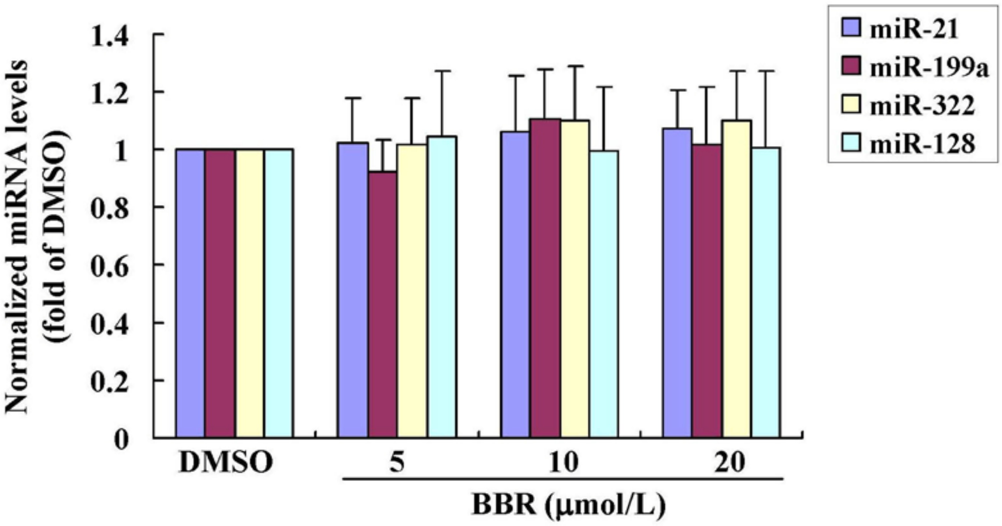

Supplementary Figure 1. Effects of BBR on the expression levels of miR-21, miR-199a, miR-322, and miR-128. Mouse primary preadipocytes were treated with DMSO or different concentrations of BBR for 72 h. After treatment, cells were harvested; the expression levels of indicated miRNAs were analyzed by real-time RT-PCR. The results were normalized to RNU6-2 and presented as fold of DMSO. Values are mean ± SD of 4 separate experiments.

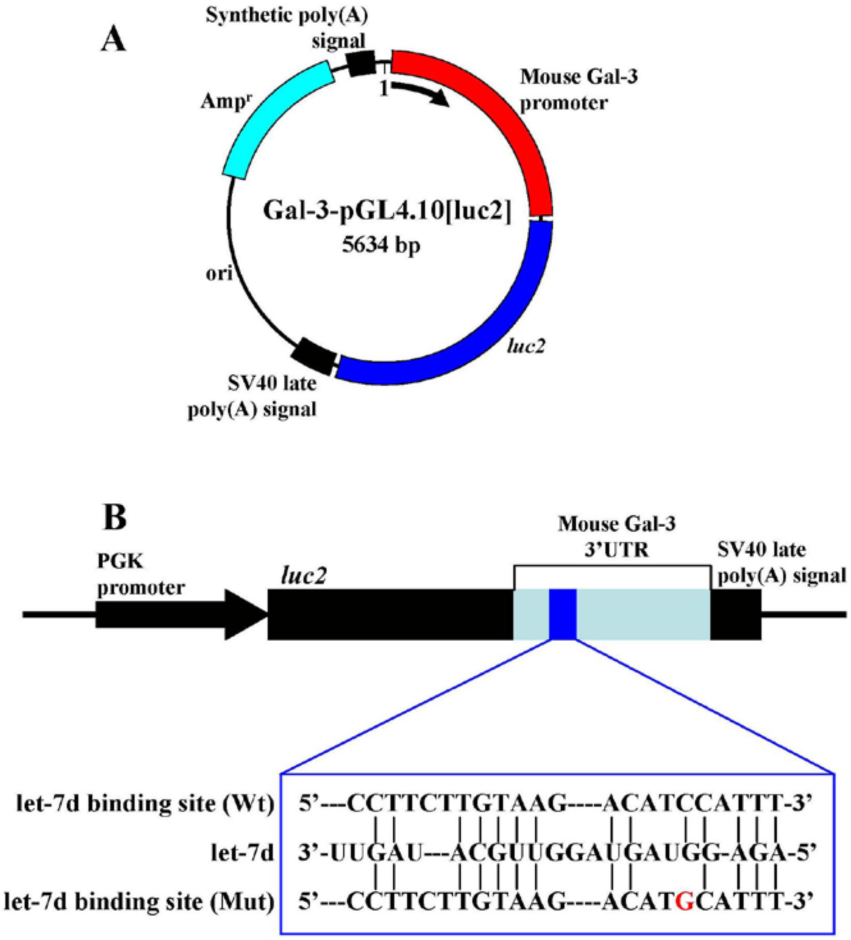

Supplementary Figure 2. Plasmid construction. (**A**) A recombinant plasmid containing the mouse Gal-3 promoter which is upstream of the *luc2* reporter gene. (**B**) Recombinant plasmids containing wild type (Wt) or a point mutation (Mut) of mouse Gal-3 3'UTR which is downstream of the *luc2* reporter gene.

Supplementary Table 1 Mouse primers for quantitative real-time PCR (5’-3’).

Gene	Forward primer	Reverse primer
Gal-3	CAGTGCTCCTGGAGGCTATC	ATTGAAGCGGGGGTTAAAGT
PPARγ	CGCTGATGCACTGCCTATGA	AGAGGTCCACAGAGCTGATTCC
C/EBPα	CGCAAGAGCCGAGATAAAGC	CACGGCTCAGCTGTTCCA
aP2	CATGGCCAAGCCCAACAT	CGCCCAGTTTGAAGGAAATC
ACTB	GGCTGTATTCCCCTCCATCG	CCAGTTGGTAACAATGCCATGT

Full blot Fig. 2A

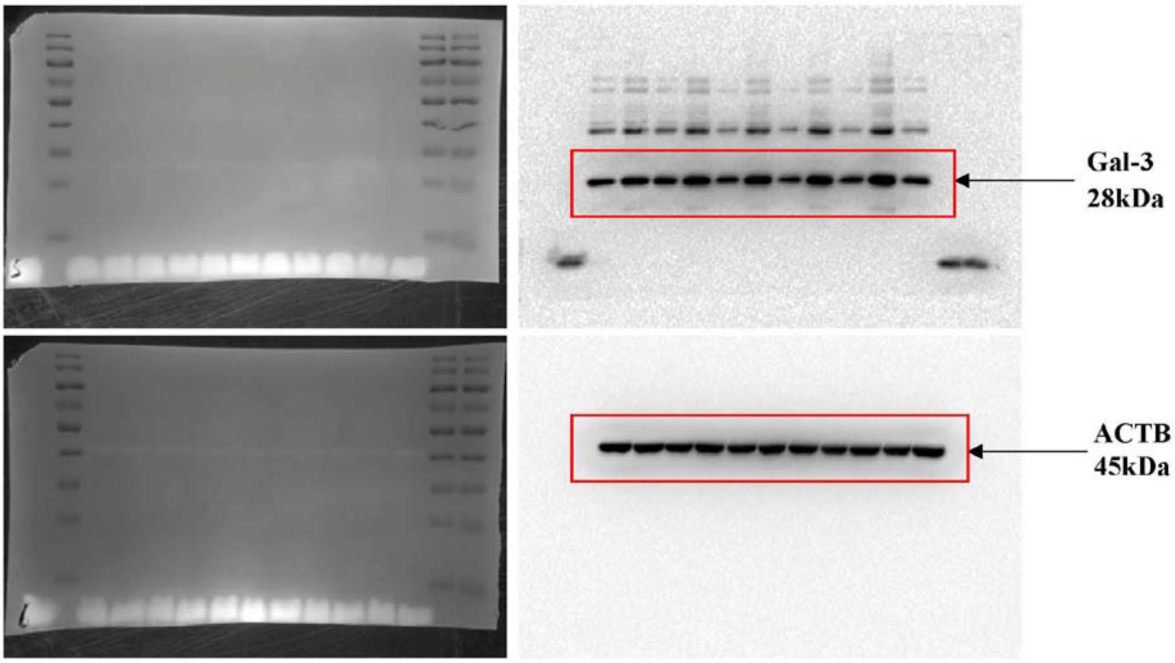

Full blot Fig. 2C

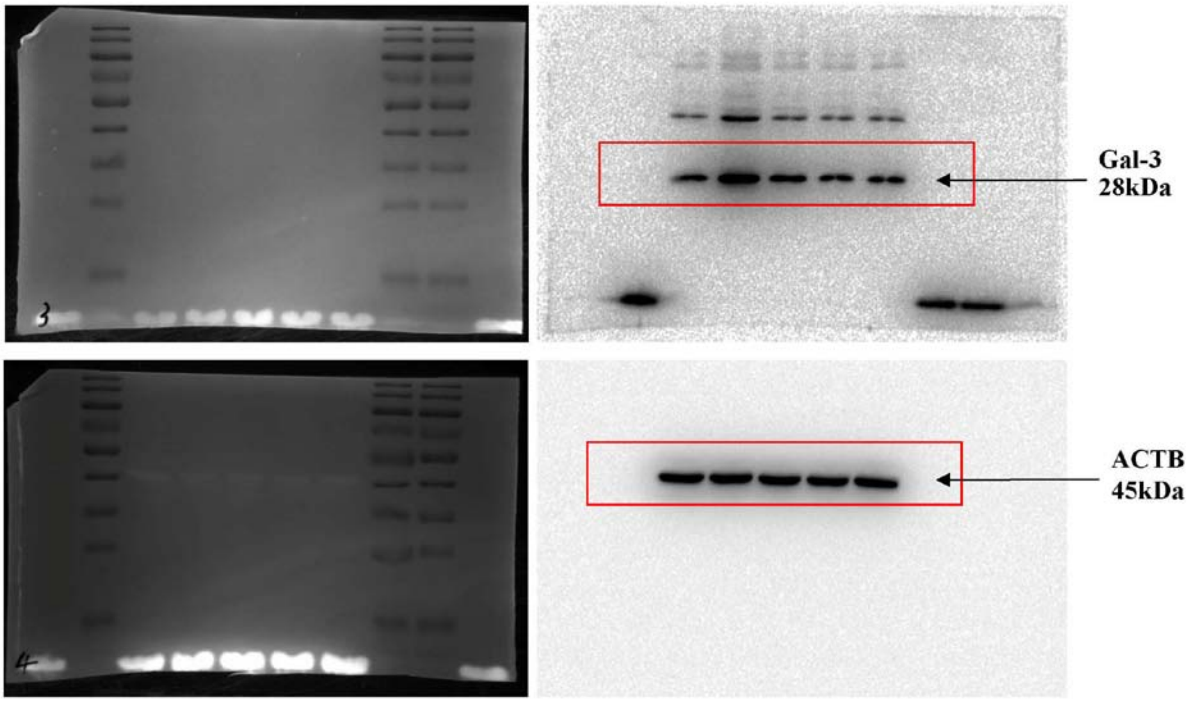

Full blot Fig. 3A

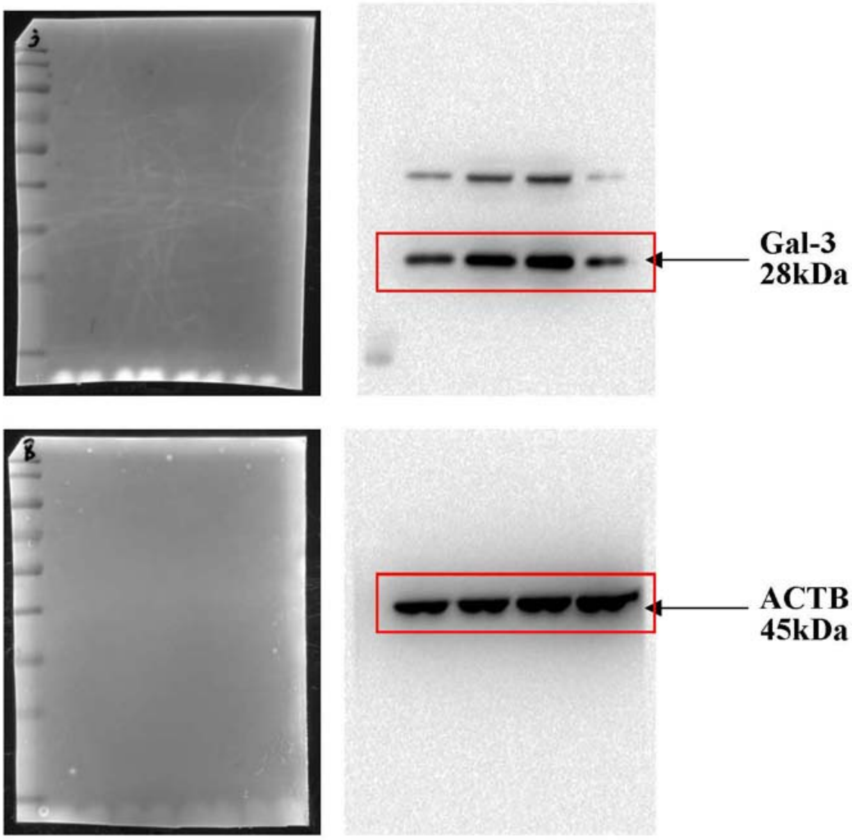

Full blot Fig. 4A

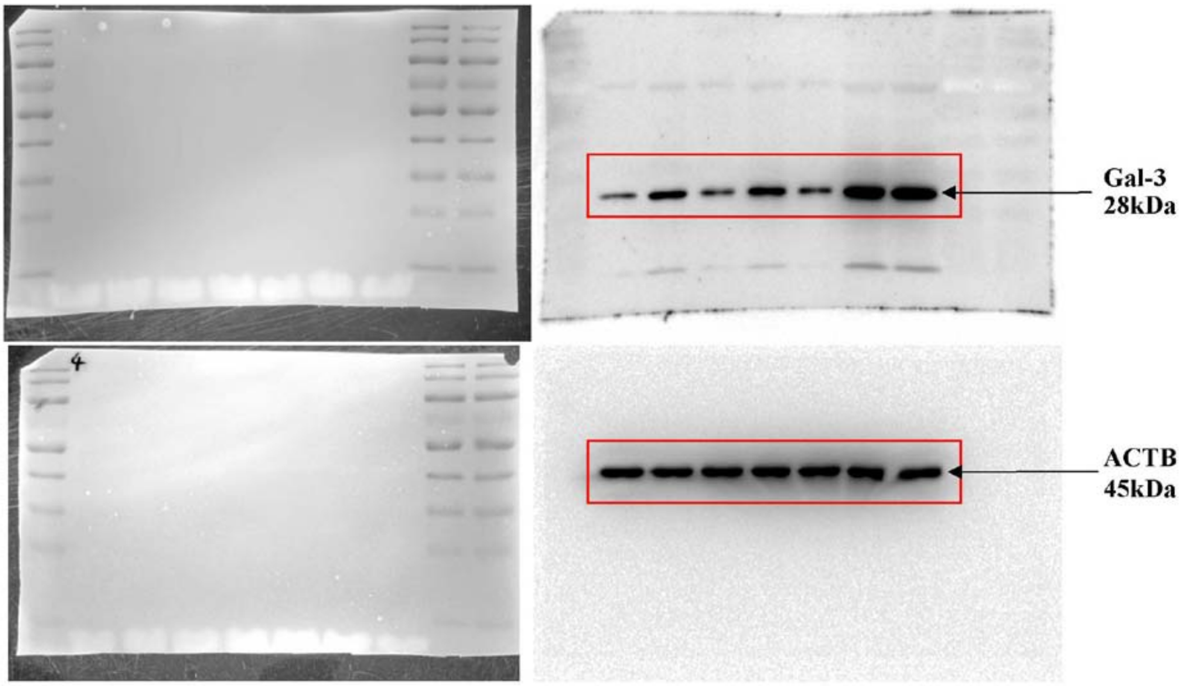

Full blot Fig. 6A

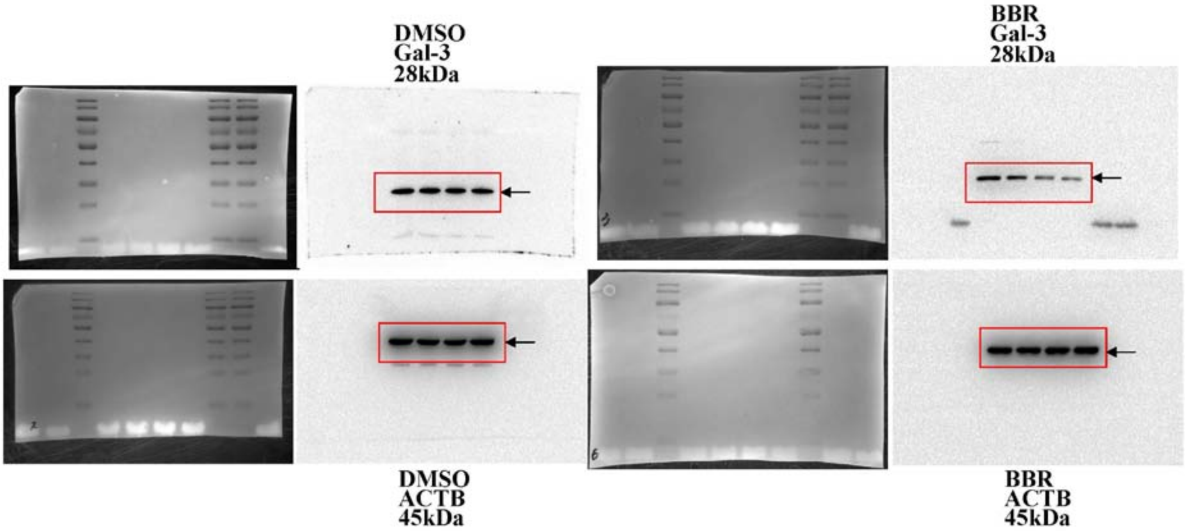

Full blot Fig. 6B

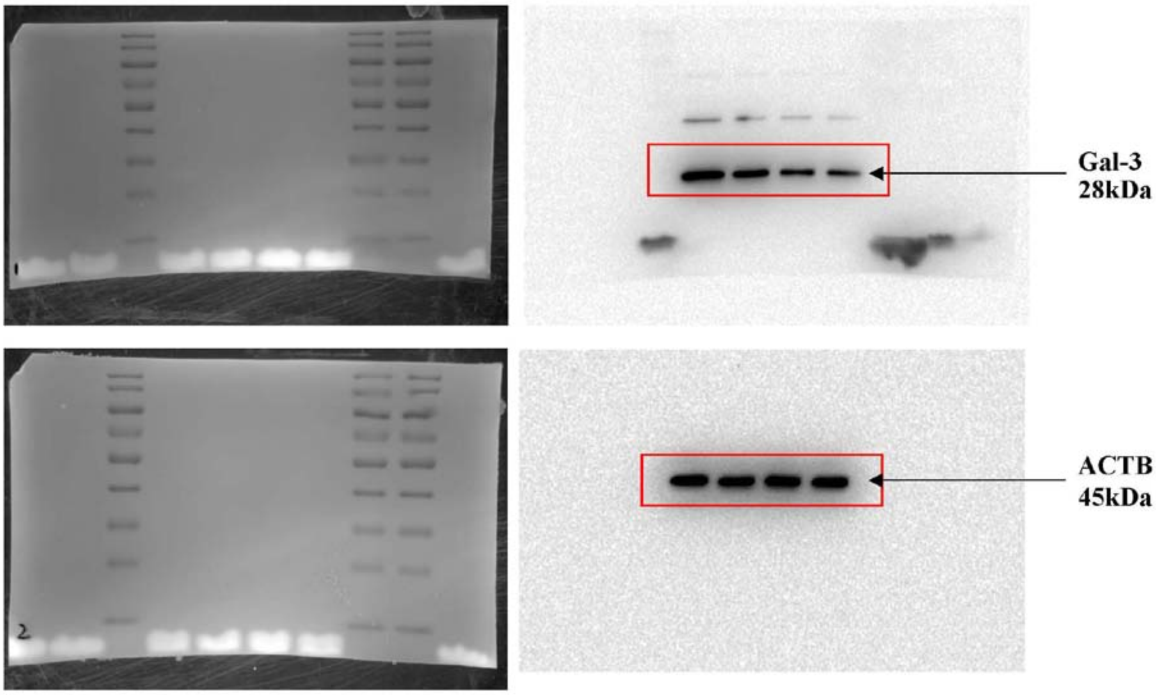

Full blot Fig. 7A

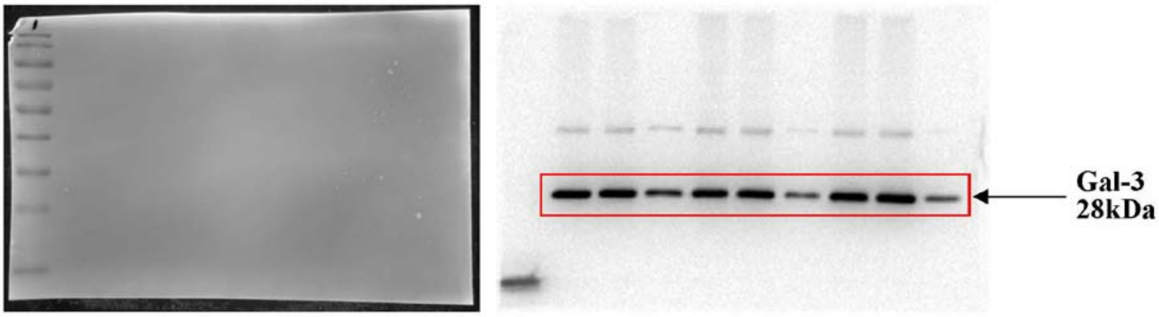

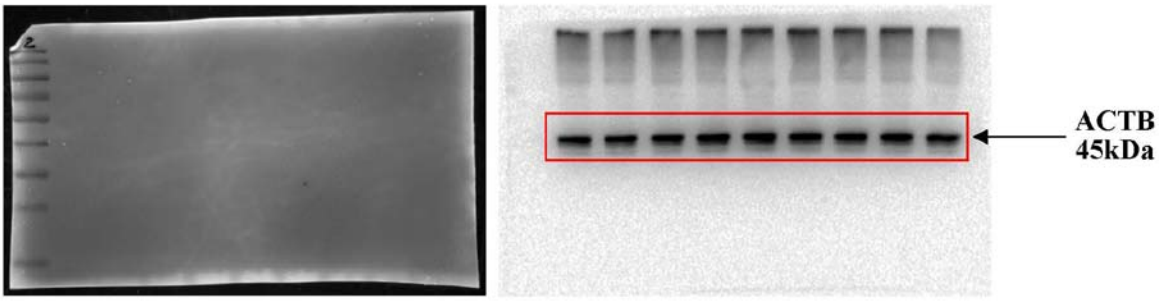

Full blot Fig. 8A

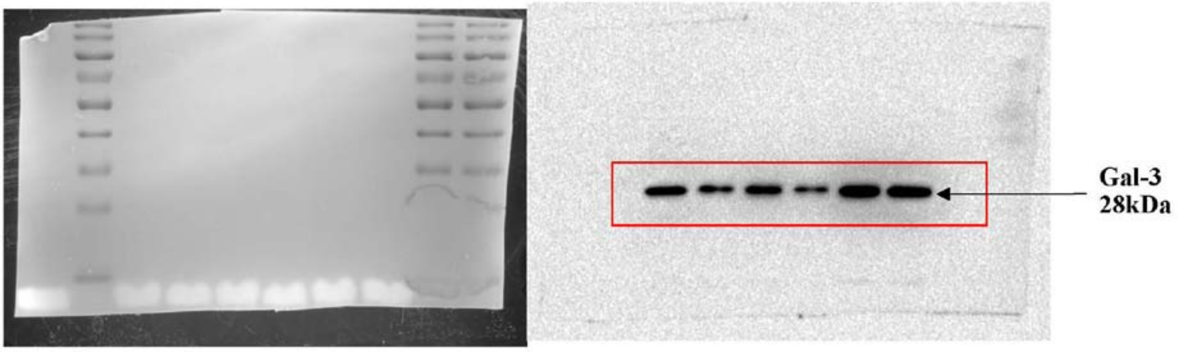

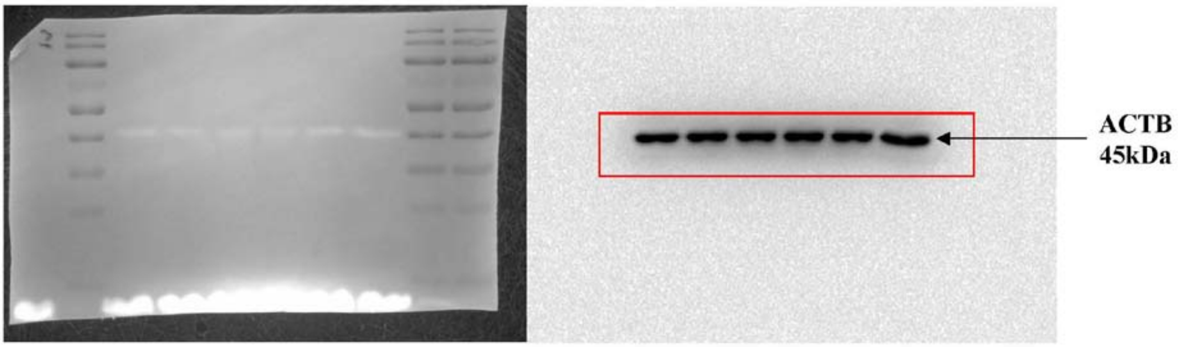

Supplement: Supplementary file 1 — Supplementary information [file 41598_2019_50103_MOESM1_ESM.pdf]
